# A prototype handheld X-ray diffraction instrument

**DOI:** 10.1107/S1600576718012943

**Published:** 2018-10-25

**Authors:** Graeme Hansford

**Affiliations:** a University of Leicester, Space Research Centre, Department of Physics and Astronomy, Leicester LE1 7RH, UK

**Keywords:** X-ray diffraction, handheld XRD devices, portable XRD devices, energy-dispersive XRD, back-reflection geometry, sample preparation, nondestructive analysis

## Abstract

A conceptual design for a handheld X-ray diffraction (HHXRD) instrument is proposed. Central to the design is the application of energy-dispersive XRD (EDXRD) in a back-reflection geometry. This technique brings unique advantages which enable a handheld instrument format, most notably, insensitivity to sample morphology and to the precise sample position relative to the instrument. For fine-grained samples, including many geological specimens and the majority of common alloys, these characteristics negate sample preparation requirements. A prototype HHXRD device has been developed by minor modification of a handheld X-ray fluorescence instrument, and the performance of the prototype has been tested with samples relevant to mining/quarrying and with an extensive range of metal samples. It is shown, for example, that the mineralogical composition of iron-ore samples can be approximately quantified. In metals analysis, identification and quantification of the major phases have been demonstrated, along with extraction of lattice parameters. Texture analysis is also possible and a simple example for a phosphor bronze sample is presented. Instrument formats other than handheld are possible and online process control in metals production is a promising area. The prototype instrument requires extended measurement times but it is argued that a purpose-designed instrument can achieve data-acquisition times below one minute. HHXRD based on back-reflection EDXRD is limited by the low resolution of diffraction peaks and interference by overlapping fluorescence peaks and, for these reasons, cannot serve as a general-purpose XRD tool. However, the advantages of *in situ*, nondestructive and rapid measurement, tolerance of irregular surfaces, and no sample preparation requirement in many cases are potentially transformative. For targeted applications in which the analysis meets commercially relevant performance criteria, HHXRD could become the method of choice through sheer speed and convenience.

## Abstract 2

Energy-dispersive X-ray diffraction implemented in a back-reflection geometry has unique characteristics, particularly its insensitivity to sample morphology. The potential to develop a handheld X-ray diffraction instrument based on this technique is explored with the help of a prototype instrument adapted from a handheld X-ray fluorescence device.

## 1. Introduction   

Energy-dispersive X-ray diffraction (EDXRD) in a back-reflection geometry is uniquely insensitive to sample morphology and to the precise distance between instrument and sample (Hansford, 2011 ▸, 2013 ▸; Hansford *et al.*, 2014 ▸, 2017 ▸). If the sample is also fine grained such that the powder-averaging criterion is satisfied, then an XRD analysis can be performed with no sample preparation at all. The back-reflection technique inherently uses low-energy X-rays, up to approximately 10 keV, and so the analysis is confined to surface layers with typical penetration depths of a few micrometres. The most basic implementation of the technique requires the same technologies as have been developed for commercial handheld X-ray fluorescence (HHXRF) devices (Bosco, 2013 ▸). The potential to develop a handheld XRD (HHXRD) instrument based on back-reflection EDXRD is explored in this paper.

Although angle-dispersive XRD is the standard configuration in the field of powder diffraction, EDXRD methods find application in niche areas primarily to take advantage of the static geometry. If a low scattering angle is chosen, say 2θ < 10°, then high-energy and therefore penetrating X-rays are necessary, allowing a transmission geometry with few constraints imposed on the experimental design. Low-angle high-energy EDXRD is therefore commonly employed in studies involving non-ambient conditions (Norby & Schwarz, 2008 ▸; Moorhouse *et al.*, 2012 ▸) and probing specific volumes within complex samples (Scarlett *et al.*, 2009 ▸). The high speed of EDXRD also enables *operando* and dynamical studies (Walton & O’Hare, 2000 ▸; Caminiti & Albertini, 1999 ▸; Zhang *et al.*, 2017 ▸). Tomographic energy-dispersive diffraction imaging [see Cernik *et al.* (2011) ▸, Espinosa-Alonso *et al.* (2010) ▸ and references therein] takes advantage of the properties of low-angle EDXRD to perform three-dimensional diffraction imaging. A key limitation of EDXRD is the relatively low resolution afforded by energy-dispersive detectors, confining studies to materials which are not too crystallographically complex. Only a small number of synchrotron EDXRD experiments that use the high resolution offered by scanning monochromators have been reported (Bourdillon *et al.*, 1978 ▸; Parrish & Hart, 1987 ▸), including the related work of the author (Hansford *et al.*, 2017 ▸). The majority of EDXRD experiments use synchrotron radiation to access high flux at high energies, but some laboratory instruments have been developed to take advantage of the static geometry (Dicken *et al.*, 2015 ▸; O’Dwyer *et al.*, 2014 ▸; Garrity *et al.*, 2007 ▸). Application of EDXRD at high angles in a reflecting geometry is much less common (Bjeoumikhov *et al.*, 2005 ▸; Sun *et al.*, 2007 ▸) because of greater overlap with XRF peaks.

A variety of portable XRD instruments have been developed, usually for specific application areas. Many are capable of combined XRD/XRF analysis, though the designs are optimized for XRD performance because this technique is inherently more technically challenging. There are currently two commercial portable XRD devices available for the general analysis of geological samples, both provided in a suitcase format. The Olympus Terra was originally derived from CheMin (Blake *et al.*, 2012 ▸), the first XRD instrument to be deployed on another planet (Bish *et al.*, 2014 ▸). It uses an innovative sample cell that ensures random grain orientations and has reduced specimen preparation requirements (Sarrazin *et al.*, 2005 ▸) so that preparation can be performed in the field. The Xplorer Planet instrument is a more recent development. At present, there are also two specialized portable instruments developed for the measurement of residual stress in alloys and retained austenite in steels: the ProtoXRD iXRD and the Pulsetec μ-X360s. In addition, several research groups have developed their own portable XRD devices for the analysis of artworks and archaeological materials (Eveno *et al.*, 2011 ▸; Cuevas *et al.*, 2015 ▸; Chiari *et al.*, 2016 ▸); these instruments have been reviewed by Nakai & Abe (2012 ▸). Portability is highly beneficial in archaeometry because curators are increasingly reluctant to allow artefacts to leave the museum or collection and transportation is not feasible in any case for large objects. All of these systems perform analyses nondestructively, of crucial importance in cultural heritage, and the instruments must typically be positioned a precise distance from the analytical spot. Several planetary XRD instruments have been proposed in addition to CheMin, as summarized by Hansford (2011 ▸). Unsurprisingly, none of the portable XRD instruments can rival the performance of standard laboratory diffractometers, but the former can nevertheless answer relevant questions relating to the properties of the material under investigation. Although proposals have been put forward for handheld XRD instrumentation in the patent literature, none have become a commercial reality and there do not appear to be any references to HHXRD in the scientific literature (other than the related publications by the author). The transformative potential of portable and handheld instrumentation is described very well by Crocombe (2013 ▸, 2018 ▸), Crocombe & Druy (2016 ▸) and Gardner & Green (2013 ▸).

The primary purpose of this study was to demonstrate the potential performance of a handheld XRD instrument based on the back-reflection EDXRD method. Experimental details are given in §2, including a description of the modifications made to an HHXRF instrument and operational details of the prototype HHXRD device. As will be seen the prototype is not truly a handheld device, but the description ‘handheld’ is justified on the basis that the instrument has been derived by relatively minor modifications to a handheld device and because it is straightforward to show that a modest evolution of the instrument design will allow handheld operation with similar or improved analytical capabilities. The methods used to process the data sets are described in §3. Results are presented in §4, including the quantification of the composition of iron-ore samples and some examples of metals analysis. Additional results, including assessment of limestone and dolomite rock samples and alloys based on Al, Ti and Ni, are reported in the supporting information. The experimental results and their implications for future work are discussed in §5, and the conclusions of this study are presented in §6.

## 2. Experimental details   

### 2.1. Modification of a handheld XRF device   

An S1 Turbo^SD^ HHXRF instrument (2008 model) was provided by Bruker Elemental (Kennewick, USA) for modification along with technical support. This instrument houses a 2.7 W Rh anode transmission X-ray tube source and a 10 mm^2^ silicon drift detector (SDD). The instrument also has a five-position primary beam filter wheel, allowing optimization of the XRF excitation conditions for a range of different sample types. In the modified instrument, the filter wheel is permanently in the ‘straight-through’ position with no filter in place.

The purpose of the modifications to the instrument was to achieve a back-reflection geometry and to ensure that geometric broadening of diffraction peaks was small relative to detector broadening (Hansford, 2011 ▸). The very compact design of HHXRF instrumentation makes modification more challenging, so the simplest possible changes were made in order to minimize the risk of damaging components or causing unexpected problems. Essentially just two modifications were made to the instrument. Firstly, the nosepiece was extended so that the sample is positioned further away from the X-ray tube source and the detector in order to achieve an approximately back-reflection geometry. The sample was moved along the projected line of the primary beam by 30 mm so that no alteration to the source was required. The dimensions of the aperture in the nosepiece were unchanged so that the same windows could be used to protect from ingress of dust or other material. The change in sample position necessitated the second modification – reorientation of the detector to face the sample. The detector was also moved closer to the revised sample position by 5.7 mm. In the modified geometry, the detector encompasses a 2θ range of 156.5–164.4° relative to the central axis of the primary X-ray beam. The modelled geometric broadening (Hansford, 2009 ▸) amounts to 0.7% compared with detector broadening of 2.5% at 5.9 keV (Hansford, 2011 ▸). Given that the broadening factors add in quadrature, geometric broadening is essentially negligible in the prototype instrument.

There are a couple of important consequences resulting from the modifications to the geometry. Firstly, no attempt was made to change the collimation of the X-ray source beam and, as a result of beam divergence and the increased source-to-sample distance, the inside surface of the Al nosepiece is exposed to X-rays. It is therefore necessary to acquire background spectra which are subtracted from the sample spectra. Secondly, the increased source-to-sample and sample-to-detector distances lead to extended data-acquisition times. Both these compromises can be addressed in a purpose-designed instrument (see the discussion in §5).

### 2.2. Instrument operation   

**Figure 1:**
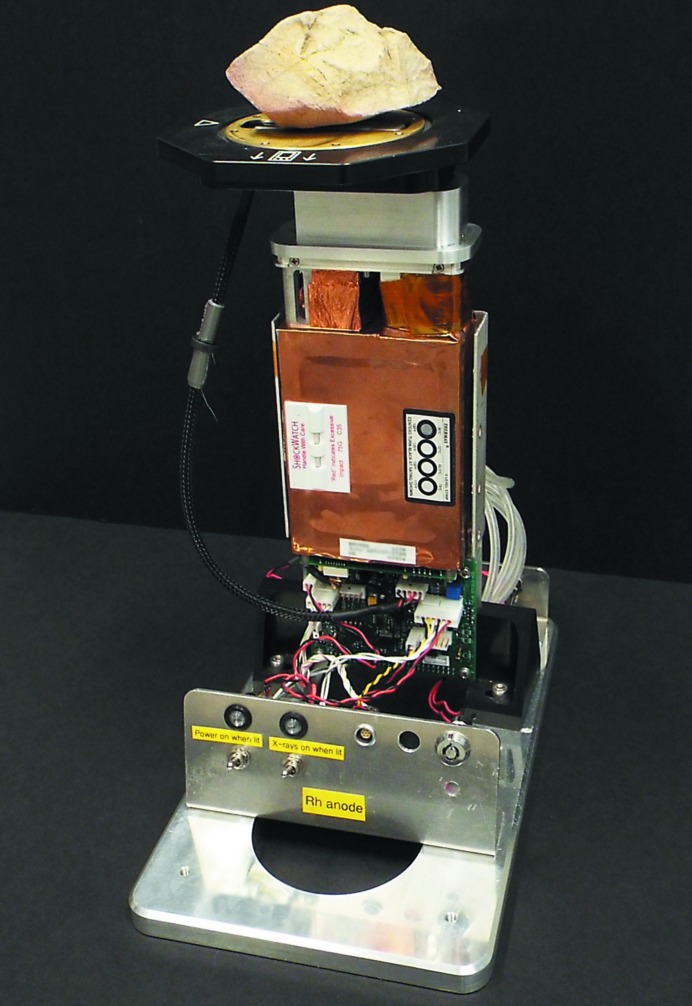
Photograph of the prototype HHXRD instrument mounted in a benchtop stand. The diaphragm pump is not shown in this image. A small sample table rests on top of the instrument nosepiece with a rock sample in place.

To achieve good signal-to-noise ratios, the prototype instrument was typically set to acquire data for 2.5 h per sample (each data set reported in this article was acquired over this period unless stated otherwise). It was therefore necessary to mount the instrument in a benchtop stand which was provided by Bruker Elemental (Fig. 1 ▸). The outer case of the original HHXRF instrument was removed and the instrument was cooled with a small fan during operation. Specimens were placed on top of a small sample table, flush with the instrument nosepiece. No sample was in place for the acquisition of background spectra; shielding (not shown in Fig. 1 ▸) prevented radiation exposure of personnel. The volume within the nosepiece was evacuated with a diaphragm pump during each acquisition, with a typical pressure of 3 mbar (300 Pa) achieved within a few minutes. This vacuum capability is a feature of the S1 Turbo^SD^ instrument (Seyfarth & Kaiser, 2013 ▸) and was intentionally retained in the design of the modified nosepiece. A window that incorporates a support grid must be in place when using the vacuum facility to avoid rupture.

All data acquired with the prototype HHXRD instrument used the maximum available X-ray tube emission current of 60 µA, while the excitation voltage was set to a value dependent on the type of sample. For example, for the analysis of iron-ore samples, an excitation voltage of 7 kV was used, a little below the 7.111 keV Fe *K* absorption edge,^1^ in order to completely suppress Fe *K* fluorescence lines that would otherwise overlay the spectrum of diffraction peaks. This ‘fluorescence suppression’ technique has been fully described in previous work (Hansford *et al.*, 2014 ▸) and is used to ensure that the spectrum of each sample is as free from overlapping XRF peaks as possible. As a shorthand, terms such as ‘Fe suppression’ are subsequently used to denote operation of the tube below the corresponding *K* absorption edge, with the voltage specified at the first occurrence.

## 3. Data-processing methods   

This section describes the data-processing methods employed for the analysis of iron-ore samples. Some minor differences in the processing of metal sample data sets are noted in §4.2. As mentioned above, a background spectrum is subtracted from each sample spectrum in order to remove the signal coming from the exposed interior surface of the nosepiece and from the vacuum window. Background spectra must be recorded using identical operating conditions but with no sample in place. All the spectra acquired by the prototype instrument have small but significant errors in the nominal energy scale. Each spectrum was individually calibrated using XRF peaks in the spectrum according to the simple linear model *E*_C_ = *a*_1_ *E*_R_ + *a*_0_, where *E*_R_ is the ‘raw’ energy as provided by the instrument, *E*_C_ is the calibrated energy, and *a*_0_ and *a*_1_ are the fitted parameters. The offset parameter *a*_0_ was consistently found to be 13.5 ± 1.0 eV while the scale parameter *a*_1_ typically differed from unity by −0.15 to +0.1%. Fitting of individual spectra was found to be essential to avoid introducing obvious artefacts when the background was subtracted.

**Figure 2:**
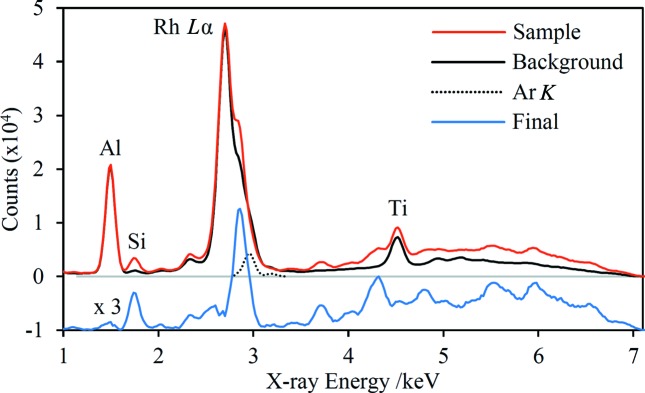
Recorded spectra showing the processing steps for one of the iron-ore spectra. The background and sample spectra are shown overlaid, and the background-subtracted spectrum is offset and multiplied by a factor of three. Simulated Ar *K* fluorescence peaks are shown to scale. Fluorescence peaks are labelled with the corresponding element and the most intense scattered Rh *L* line is also labelled. The Al peak is due to to exposure of the interior surface of the nosepiece, while the Ti peak is primarily the result of small amounts of this element in the vacuum window (probably the support grid). The strong peak at ∼2.9 keV in the background-subtracted spectrum is diffraction-enhanced Rh *L*β.

The X-ray tube source emits Rh *L* characteristic lines in the range of approximately 2.4–3.1 keV. These lines undergo Compton and Rayleigh scattering by the sample and are usually the strongest features in the recorded spectra, potentially masking diffraction peaks in this spectral range. Puzzlingly, the scattered Rh *L* lines have greater intensity in the background spectra than in many of the sample spectra, leading to negative-going peaks in the background-subtracted spectra. A discussion of this phenomenon can be found in the supporting information. Fig. 2 ▸ shows the Fe-suppression background spectrum along with one of the iron-ore spectra before and after subtraction of the background. Individual Rh *L* lines may also be enhanced by overlap with a diffraction peak, as seen in Fig. 2 ▸. These enhancements are not considered reliable information because small changes in the lattice parameter will cause an angular shift of the Debye–Scherrer ring, potentially changing the overlap with the detector and leading to a significant alteration in the peak intensity. This spectral range is therefore generally ignored in the analysis, especially when attempting phase quantification. Furthermore, the X-ray tube has relatively low *Bremsstrahlung* output in the range 3.0–3.4 keV because of strong absorption by the Rh anode above the Rh *L* absorption edges. Consequently, the useful energy range for quantification purposes typically lies upwards of ∼3.3 keV.

The presence of Ar in air generates Ar *K* fluorescence peaks near 3 keV in the background spectra. These signals are largely absent in the sample spectra because there is a negligible X-ray path length in air when a sample is in place. A correction is made by subtracting simulated Ar *K* peaks from the background spectrum (see Fig. 2 ▸); the strength of these peaks is estimated by assessing the effect on the final sample spectra and is therefore somewhat subjective. However, this issue is a minor one since the Ar *K* peaks lie within the range that is usually neglected.

An analytical model of the instrument response was fitted to the iron-ore data in a non-linear least-squares fitting routine in order to identify and quantify the mineral constituents. The model calculates a source spectrum and applies fluorescence and diffraction equations (Hansford, 2009 ▸) according to the sample composition. Rayleigh scattering is calculated using the differential cross section given by Markowicz (2002 ▸) and integrating over the sample depth according to the formulation described by Jenkins *et al.* (1995 ▸), replacing fluorescence with scattering. The solid angles of the primary beam and the X-ray flux reaching the detector are accounted for along with attenuation by the vacuum window. The detector is modelled *via* a response matrix function which is a combination of the energy redistribution model developed for CCD22 charge-coupled devices (Burrows *et al.*, 2005 ▸) and the quantum efficiency of the SDD. Variation of parameters within the solid angles of the beams is not accounted for and the analytical model is therefore most accurate for geometries with small X-ray-beam solid angles. The primary reason for using this model is speed. A single calculation is typically completed within a second, allowing the model to be incorporated into non-linear least-squares fitting which requires execution many times over. The analysis is performed within IDL v6.4 and uses the MPFIT function (Markwardt, 2009 ▸).

The calculation of the X-ray source spectrum follows the formulation by Ebel (1999 ▸, 2003 ▸) for a reflection tube because the equivalent function for a transmission tube is not available in the literature. The differences between the two are expected to be major, in terms of both total emission and the variation of intensity as a function of energy. To overcome this problem, a polynomial correction was applied to the source calculation to achieve good agreement between the experimental and modelled spectra of a pure hematite (Fe_2_O_3_) sample. Although calibration of the model in this way primarily corrects the errors in the calculation of the source intensity, in fact the whole chain of calculations affecting the final intensity profile is encompassed. This process could be viewed as a simple way to train the model with a sample representative of the intended analytes.

## 4. Results   

### 4.1. Iron ore   

Eleven iron-ore samples from working mines were used to test the ability of the prototype HHXRD instrument to identify and quantify the mineralogical composition. Five of the samples were provided by ArcelorMittal and come from the Tokadeh mine in Liberia (four samples from distinct stratigraphies with differing compositions) and from Baffinland in Canada (one sample). The remaining six samples are Australian standard certified reference materials (ASCRMs) from Western Australia, provided by CSIRO (the Commonwealth Scientific and Industrial Research Organisation). All of the samples were received as powders and were analysed either as pressed-powder pellets (ArcelorMittal samples) or in XRF powder cups obtained from Analysco. For the latter, the instrument probes the sample through a 4 µm polypropyl­ene film; attenuation of X-rays at 3.3 keV for a double pass through this film is just 4%, rapidly approaching zero at higher energies, but the effect has been included in the model nevertheless.

Each data set was prepared as described in §3 and the energy range 3.25–7.1 keV was selected for fitting. The sample is initially assumed to consist of equal amounts of four minerals: hematite, goethite [FeO(OH)], magnetite (Fe_3_O_4_) and quartz (SiO_2_) (subsequently referred to as the ‘primary’ minerals). Minor amounts of elements other than those in the primary minerals may be present in the sample, incorporated either *via* solid solutions or as additional minerals. It was necessary to include the *K*-series XRF peaks of K, Ca, Ti, Cr and Mn in the fluorescence calculation of each fit in order to derive reliable mineral quantification. One of the Tokadeh mine samples showed relatively intense Ca *K* XRF peaks and the Ca *K*α peak was excluded from the fit in this particular case. Other potentially interfering elements, such as Sc, V and elements with *L*-series lines in this spectral range, generally have very low abundances in geological samples and were excluded.

A total of ten parameters were varied in each fit: three relating to the mineral amounts (one less than the number of minerals since they were constrained to sum to 100%), five specifying the amounts of K, Ca, Ti, Cr and Mn in the fluorescence calculation, a scaling factor in the Rayleigh scattering calculation, and the overall exposure time. In principle, Rayleigh scattering can be calculated for a known composition with no free parameters, and of course the exposure time is a known quantity, but it has been found necessary to allow these two parameters to vary in order to achieve good agreement between the data and the fitted spectra; this point is discussed further below. Minerals and elements for which the estimated error was greater than the fitted amount were excluded and the fit was run again.

**Figure 3:**
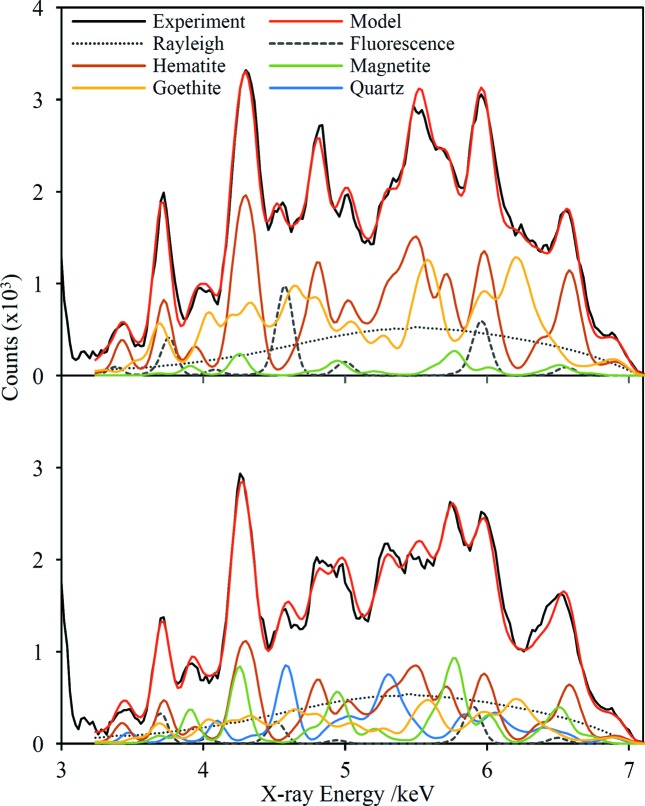
The prototype HHXRD spectra for two of the iron-ore samples alongside the model fits. The contribution to the overall fit from each mineral and the fluorescence and Rayleigh scattering contributions are also shown.

**Figure 4:**
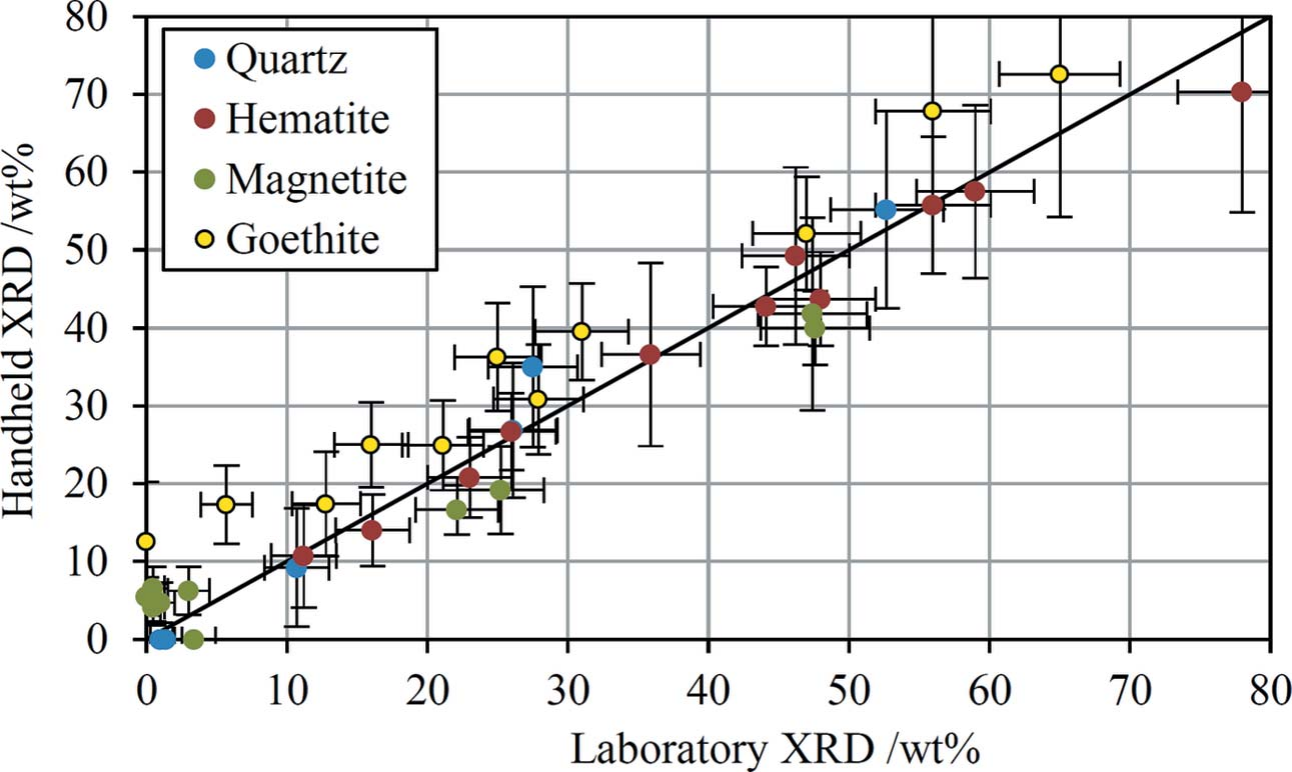
The iron-ore phase quantifications provided by the HHXRD prototype are plotted against the laboratory results. The solid line is the 1:1 correspondence line for reference; it is not a fit to the data points. The error bars correspond to the error estimates reported in Table 1 ▸.

Fig. 3 ▸ shows the fitted spectra overlaid on the experimental data for two of the samples, along with the decomposition of the fit into its different components. These two examples are entirely representative and the level of agreement between the fit and the data is very similar for all 11 samples. To assess whether the HHXRD prototype can quantify the mineralogical composition of iron ores, the results are compared with independent analyses performed with standard laboratory diffractometers. The ASCRMs have published compositions (Knorr & Birch, 2013 ▸) while the ArcelorMittal samples were sent to a commercial laboratory (James Hutton Ltd) for analysis. The comparison is shown in Table 1 ▸ and graphically in Fig. 4 ▸. It is clear from the figure that the laboratory and HHXRD results correlate very well and that the prototype instrument provides genuine quantification of the four analysed minerals in these 11 samples. The absolute difference in the amounts quantified by the two methods is 4.6 wt% averaged across all four minerals but excluding data points ≤1 wt% by both methods. The points corresponding to hematite and quartz in Fig. 4 ▸ appear to scatter randomly about the 1:1 correspondence line, whereas all the goethite points lie above this line and all the magnetite points, except where the laboratory analysis shows <5 wt%, lie below the line. The reason for the biases for these two minerals is not entirely clear. Normalization of the four primary minerals to 100% in the HHXRD analysis necessarily leads to overestimation if other phases are present in the sample. But the fact that the over- or underestimation of specific minerals is consistent between samples points to some form of model inaccuracy as the underlying cause. Goethite has the lowest crystal symmetry (orthorhombic) of the four minerals and consequently has the ‘smoothest’ least-structured diffraction profile. It is possible that the diffraction intensity due to to minor unidentified minerals and amorphous materials, and even some of the Rayleigh-scattered intensity, may be partly absorbed into the goethite quantification. Interestingly, magnetite has the highest crystal symmetry (cubic) and is underestimated by the HHXRD quantification. A separate issue for magnetite is that the fits of HHXRD spectra often report a statistically significant detection of typically ∼5 wt% even when the laboratory data indicate ≤1 wt%.

Table 1 ▸ reports the laboratory determination of the amounts of minerals other than the primary four and, for the ASCRMs, the amorphous content. For the HHXRD fits, the Rayleigh scattering scale factor and the total exposure time are also reported. There are good reasons to expect some correlations between these various parameters. For example, minerals that are present in the sample but not included in the HHXRD fits contribute diffraction intensity to the spectrum and, especially if they have low symmetry and so relatively featureless diffraction profiles, may lead to a higher Rayleigh scattering scale factor. Samples with a higher proportion of light elements than captured by the quantification of the primary minerals may be expected to have a lower fitted exposure time since lighter elements have lower X-ray scattering cross sections. Plots of the parameters in the last four columns of Table 1 ▸ (see the supporting information) show a good negative correlation between the amorphous fraction and the fitted exposure time, and a positive correlation between the amorphous fraction and the Rayleigh scattering scale factor. Necessarily, there is a negative correlation between the exposure time and the Rayleigh scale factor. The overestimate of goethite in the HHXRD data correlates with the amount of ‘other’ minerals, with the amorphous fraction and with the sum of these two parameters, but none of the correlations are particularly tight. The study of the ASCRMs (Knorr & Birch, 2013 ▸) showed that the amorphous fraction is Si- and Al-rich, and low in Fe, complicating the interpretation. To some extent at least, the expectations described above are borne out. However, there are insufficient data to reach firm conclusions regarding the biases in the HHXRD results and further work is warranted to disentangle the various effects.

### 4.2. Analysis of metals   

Samples of standard-grade alloys were obtained from a variety of sources, including an alloy check sample kit from PMI Analytical (commercially available for checking alloy identification by HHXRF instruments). The steps taken to process the spectra of metal samples were as described in §3, namely calibration of the energy scale and subtraction of the background spectrum, including a correction for Ar *K* fluorescence. The calculation of the X-ray tube emission spectrum was calibrated at each excitation voltage against spectra of the NIST 640e Si diffraction standard (Small & Watters, 2015 ▸) by applying a polynomial correction function. Whereas the majority of geological samples exhibit heavy overlap of diffraction peaks, the major phases in metals have relatively sparse diffraction patterns. The prototype HHXRD instrument therefore yields essentially resolved diffraction spectra with much lower levels of peak overlap. Consequently, the baseline of each spectrum, primarily caused by scattering of the X-ray tube continuum emission, could be separately fitted with a polynomial function and subtracted. The final spectra therefore consist essentially of peaks due to to diffraction and fluorescence. All of the spectra shown in this section have been baseline-subtracted in this way, unless otherwise noted, for the energy range above approximately 3.3 keV. For samples containing more than one detectable phase, quantification has been attempted by model fitting but the results have not generally been independently verified.

#### 4.2.1. Copper alloys   

**Figure 5:**
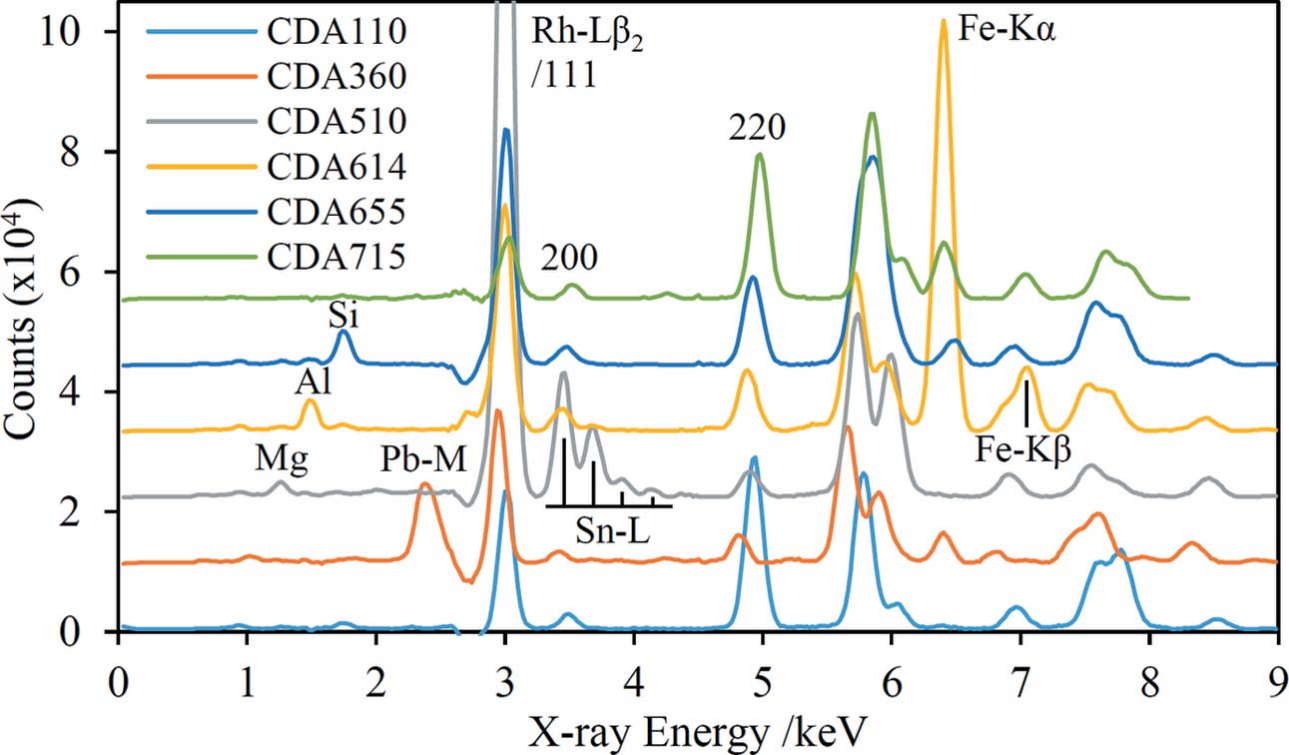
The prototype HHXRD spectra of the six Cu alloy samples, five acquired using Cu suppression and the CDA715 spectrum acquired using Ni suppression. The latter spectrum has been multiplied by a factor of 1.25 to approximately compensate for the lower X-ray tube *Bremsstrahlung* output. XRF and the first three diffraction peaks are labelled with the corresponding elements and Miller indices, respectively. The 111 peak is enhanced by overlap with Rh *L*β_2_ in some cases.

A set of six standard-grade Cu alloys with a wide range of alloy compositions (Table 2 ▸) were analysed. The X-ray tube was operated at 8.95 kV in order to suppress Cu *K* fluorescence and the acquired spectra are shown in Fig. 5 ▸. The alloys CDA614 and CDA715 contain significant amounts of Fe and Ni, respectively, and additional spectra were acquired for these samples at 7.0 and 8.3 kV excitation voltages to suppress the corresponding *K*-series fluorescence peaks. For the CDA715 sample in particular, operating at the lower voltage leads to a ‘cleaner’ EDXRD spectrum with more diffraction peaks not overlapped by XRF peaks; this Ni-suppressed spectrum is shown in Fig. 5 ▸.

The data from the HHXRD prototype show clearly that all of the Cu alloy samples are dominated by the α-Cu face-centred cubic (f.c.c.) phase, as expected (Davis, 2001 ▸). It has been possible to identify a second phase for only one of the samples, CDA360. Three resolved, albeit weak, peaks at 4.25, 5.20 and 7.94 keV are assigned as the 200, 211 and 321 diffraction lines of the β′ phase of the Cu/Zn system, *i.e.* ordered CuZn (Davis, 2001 ▸). Other predicted peaks of this phase are overlapped by stronger features in the spectrum. The model fit yields ∼4 wt% for the β′ phase, but this amount is quite sensitive to small changes in details of the spectral processing such as baseline fitting and subtraction. This alloy also contains 2.5 wt% Pb, which has extremely low solubility in Cu and is expected to precipitate as a distinct phase. Although the addition of f.c.c. Pb to the model fit leads to a modest decrease in the χ^2^ goodness-of-fit parameter (Press *et al.*, 2007 ▸), no distinct diffraction features are discernible in the spectrum and detection of this phase cannot be claimed.

**Figure 6:**
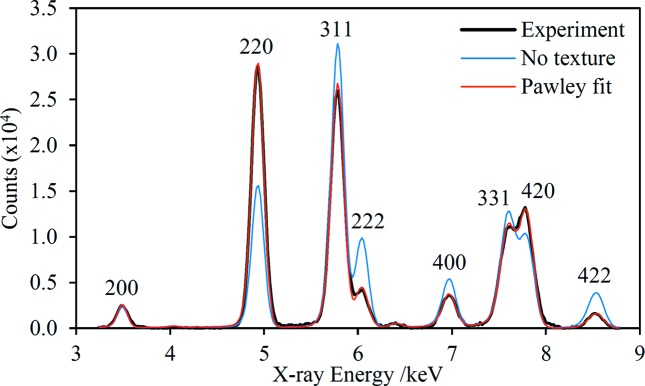
The CDA110 Cu-suppression spectrum with two model fits, one with no crystallographic texture and one Pawley fit. There is a very weak Fe *K*α XRF peak at 6.4 keV.

**Figure 7:**
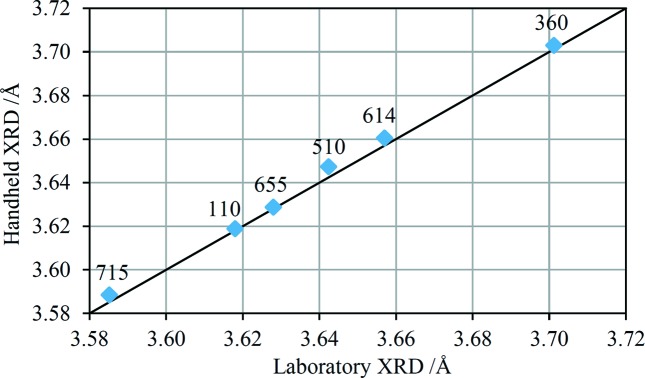
The α-Cu lattice parameters for the six Cu alloy samples derived by the HHXRD prototype compared with laboratory diffractometer measurements. The solid line shows a 1:1 correspondence for reference. The labels identify each alloy by the last three digits of the alloy designations.

The EDXRD spectrum of the CDA110 sample in the range 3.25–8.8 keV is shown in Fig. 6 ▸ along with two different model fits. In one of the fits it is assumed that the sample has no crystallographic texture – there are clearly quite significant discrepancies in the model peak intensities relative to the experimental data, providing good evidence that this sample does in fact exhibit quite strong texture. The second model fit is a Pawley-type fit in which the intensity of each peak in the spectrum is a free parameter (Pawley, 1981 ▸); the model can reproduce the experimental data with high fidelity as long as the peak intensities are correctly accounted for. This type of fit has been performed for each Cu alloy in order to extract the most accurate lattice parameters for comparison with values measured independently using laboratory XRD (Fig. 7 ▸). Overall, there is very good agreement between the two sets of measurements, with an average discrepancy of 0.0026 Å. There is a systematic offset between the two sets of measurements, with the HHXRD values consistently higher. The origin of the offset is not known but could reflect errors in either set of measurements, such as a systematic error in the energy-scale calibration of the HHXRD spectra.

#### 4.2.2. Steel alloys   

A broad set of standard- and research-grade steel alloys were used to test the capabilities of the HHXRD prototype for this ubiquitous material. The results presented here are highlights chosen to illustrate specific conclusions derived from all the data. Data sets were acquired using Fe and/or Cr suppression, the latter especially for stainless steels which have relatively high Cr content. Although suppression of the Cr *K* fluorescence peaks leads to ‘cleaner’ spectra that are usually free of XRF peaks in the useful energy range of 3.3–5.9 keV, only three austenite and/or two ferrite diffraction peaks are observable in this range. Each phase has an additional peak near 3 keV; these peaks overlap if both phases are present.

For all of the iron-based samples, the only crystallographic phases detected by the prototype instrument are austenite, ferrite and/or martensite. No minor phases, such as carbide precipitates, were observable in any case. Ferrite and martensite have closely related crystal structures with very similar diffraction patterns, particularly at the resolution afforded by the handheld instrument. The following text refers to ferrite, and the corresponding diffraction peaks in the figures have been labelled with ferrite Miller indices, but it should be understood that the samples may contain martensite or both phases. The possibility of distinguishing ferrite and martensite using HHXRD is addressed in the supporting information.

**Figure 8:**
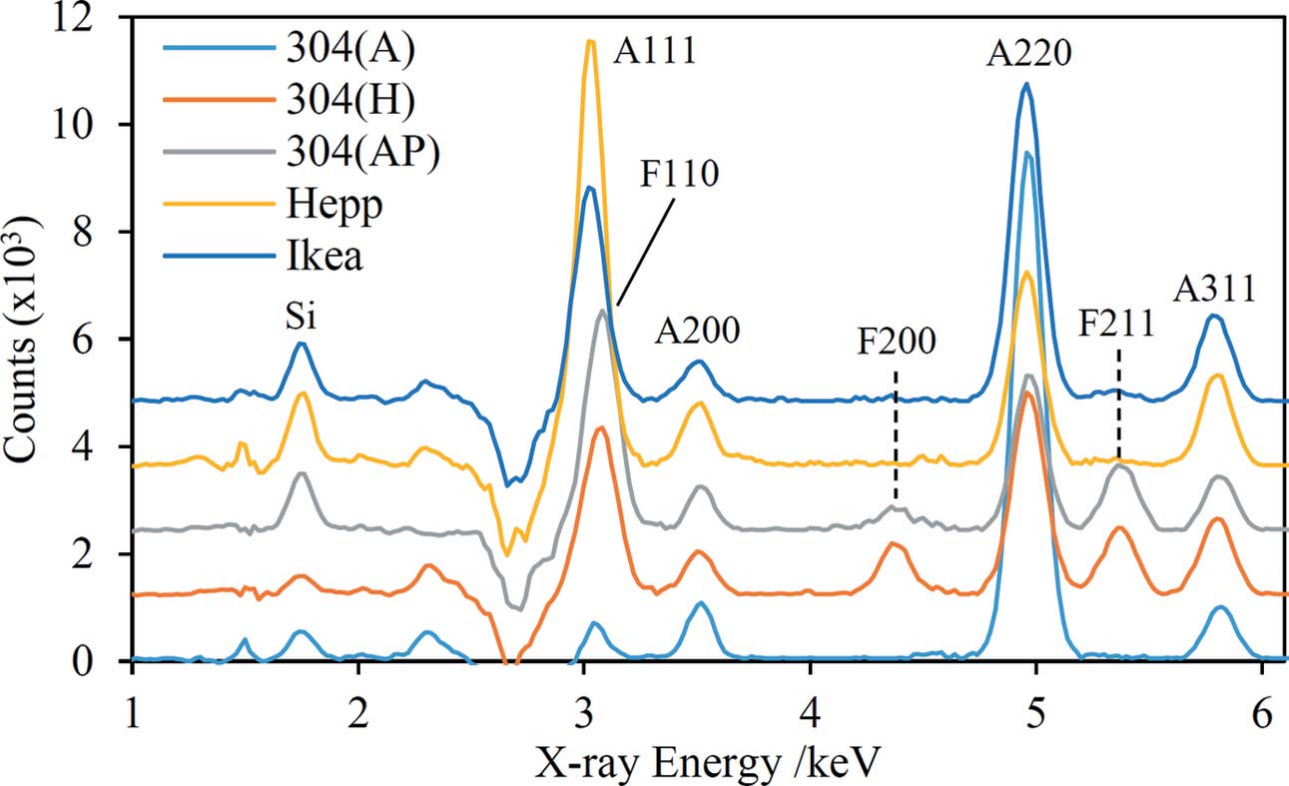
The Cr-suppression spectra for five 304 stainless steel samples. The designations in parentheses are as follows: A – annealed, H – hard, AP – annealed and pickled. The 304(A) and 304(H) samples were supplied by Goodfellow. Hepp and Ikea are the manufacturers of 304-grade household spoons; these objects were analysed on their convex surfaces. The diffraction peaks are labelled with their Miller indices and a preceding letter to indicate austenite (A) or ferrite (F).

Fig. 8 ▸ shows the Cr-suppression data sets for a set of five 304-grade steel samples (UNS S30400), the most widely used grade of stainless steel. These samples come from different suppliers and/or have had different heat treatments (see the figure caption for full details). Two of the samples are household spoons with unknown treatment histories; they were identified as 304SS by HHXRF. 300-series steels are austenitic, and indeed relatively strong austenite diffraction peaks are seen in all five spectra. The relative intensities of the austenite peaks differ quite significantly between the samples, indicating marked texture differences. Furthermore, two of the samples clearly contain a significant proportion of ferrite, estimated to be ∼30 wt% for the 304(H) sample, though it should be noted that the presence of texture interferes with the accuracy of the quantification. The HHXRD instrument detects quite major crystallographic differences between these samples despite their identical chemistries, reflecting differences in treatment histories.

**Figure 9:**
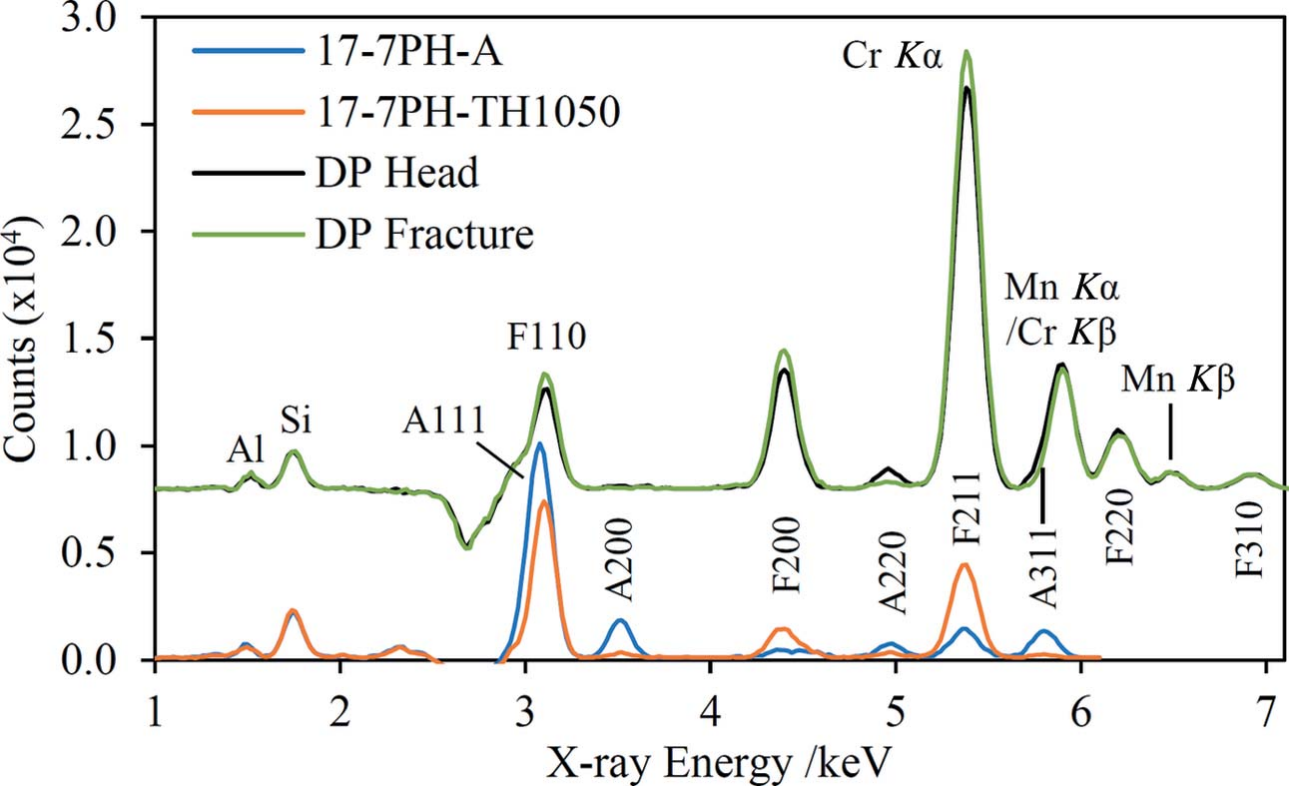
Pairs of spectra illustrating changes due to heat treatment of a precipitation-hardening steel (17-7PH, Cr-suppression data) and destructive mechanical testing of a duplex steel (Fe-suppression data). ‘Head’ denotes the unaffected part of the duplex steel sample and ‘Fracture’ denotes the part subjected to tensile stress. Diffraction peaks are labelled with Miller indices and a preceding letter to indicate the phase.

The HHXRD instrument may be particularly suited to the detection of changes in the crystallographic parameters of metallic samples or differences between similar samples; two examples are shown in Fig. 9 ▸. The first example is a precipitation-hardening steel, 17-7PH grade (UNS S17700), which was supplied in condition A (mill annealed). Part of this sample was then subjected to the TH1050 heat treatment (Chandler, 1995 ▸). Comparison of the Cr-suppression data sets for the two samples shows a clear change in the composition, namely loss of austenite and an increase in ferrite/martensite after heat treatment consistent with the expectation of martensite formation upon precipitation hardening (Pollard, 1993 ▸). The second example shows the effect of destructive tensile testing on a bone sample of research-grade duplex steel. In this case, the change in the EDXRD spectrum is more subtle but also shows a loss of austenite and a corresponding increase in ferrite, consistent with laboratory diffractometer measurements conducted by Tata Steel UK.

An important question in the HHXRD analysis of steels is the sensitivity of the technique to low levels of austenite in ferritic/martensitic steels (Magner *et al.*, 2002 ▸) and *vice versa*, and some of the data sets in Figs. 8 ▸ and 9 ▸ give insight into this issue. For example, the Ikea spectrum in Fig. 8 ▸ has a weak ferrite 211 peak and model fitting suggests 3.1 wt% ferrite in this austenitic steel. In fact, the Hepp spectrum has an even weaker peak at the same energy, with model fitting yielding 2.0 wt% ferrite. Low levels of austenite are seen in the duplex steel spectra in Fig. 9 ▸. Although not obvious at the scale plotted, there is a distinct austenite 220 peak at 4.95 keV in the Fracture spectrum, as well as a stronger peak in the Head spectrum. Model fits yield 7.1 and 0.7 wt% austenite for the Head and Fracture samples, respectively. Sensitivity to low amounts of austenite is greater for the duplex steel because the low amount of Cr allows a higher tube voltage to be used, yielding improved signal-to-noise ratios. On the basis of these results, in Cr-suppression data sets a lower limit of detection of ∼2 wt% for both ferrite and austenite is probably realistic, while the detection limit may be improved to <1 wt% for steels with low Cr content. The detection of low levels of austenite relies on observation of the 220 peak near 5.0 keV, and it is therefore important that the steel contains negligible amounts of Ti and V because of overlap with the Ti *K*β and V *K*α fluorescence peaks. However, none of the ferritic or martensitic steels tested as part of this work suffered this interference.

### 4.3. Texture measurement   

**Figure 10:**
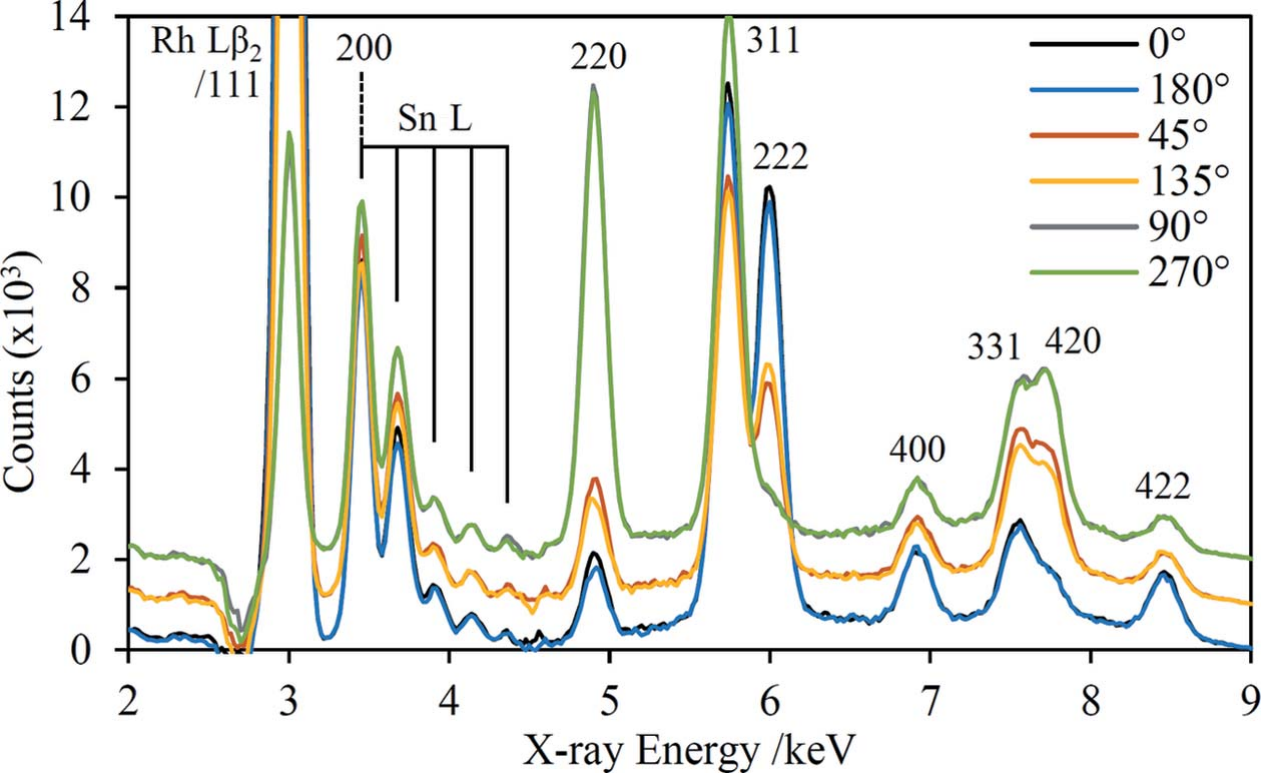
Cu-suppression spectra of the CDA510 Cu alloy sample taken at a series of φ angles, shown in the legend. No baseline subtraction has been performed and each spectrum was acquired over one hour. The spectra have been offset on the vertical axis in pairs, chosen on the assumption of biaxial texture symmetry. The diffraction peaks have been labelled with their Miller indices; 111 is enhanced by overlap with the Rh *L*β_2_ tube emission line.

To exemplify the capability of the prototype HHXRD instrument in texture analysis, several spectra were acquired with the CDA510 sample mounted in different orientations relative to the instrument. This particular sample was chosen because it shows relatively pronounced texture. Fig. 10 ▸ shows a series of spectra for rotation of the sample about the axis normal to its surface (φ rotation). For some of the diffraction peaks there are marked changes in intensity, notably 220 and 222. The spectra are shown in the figure offset in pairs at related angles, and the similarity within each pair is consistent with biaxial texture symmetry which typically results from rolling of sheet metal (Hu, 1974 ▸).

**Figure 11:**
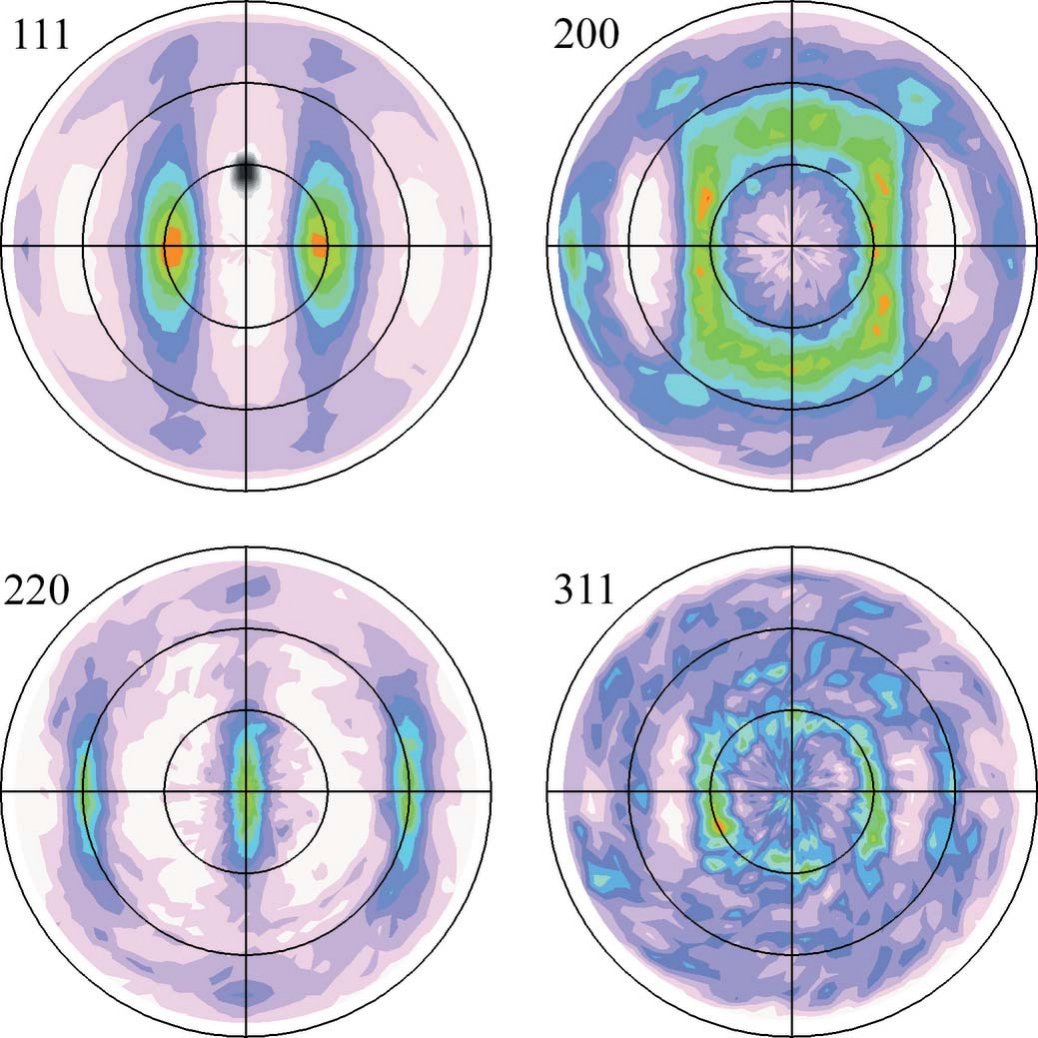
Pole figures measured for the CDA510 sample using a PANalytical Empyrean diffractometer; the Miller indices are shown adjacent to each pole figure. Diffraction peaks were background-corrected and the data have also been corrected for the effects of defocusing (Randle & Engler, 2000 ▸). The black circles lie at 30° intervals in χ and the data extend up to χ = 85°. The φ = 0° axis extends from the centre of each pole figure to the right, and the positive φ direction is anticlockwise. The approximate area encompassed in a single HHXRD measurement is shown overlaid on the 111 pole figure as a dark spot at φ = 90°.

The texture of the CDA510 sample has also been measured using a PANalytical Empyrean laboratory diffractometer fitted with a five-axis cradle. The pole figures for four of the diffraction peaks are reported in Fig. 11 ▸, confirming the biaxial symmetry. The dark shaded area marked on the 111 pole figure shows approximately the angular region interrogated in a single HHXRD measurement when the sample rests horizontally on the nosepiece in the φ = 90° position. This area is off-centre, corresponding to a non-zero χ tilt angle, because of the asymmetric diffraction geometry of the prototype instrument. This asymmetry enables detection of the biaxial symmetry by changing the φ angle only.

**Figure 12:**
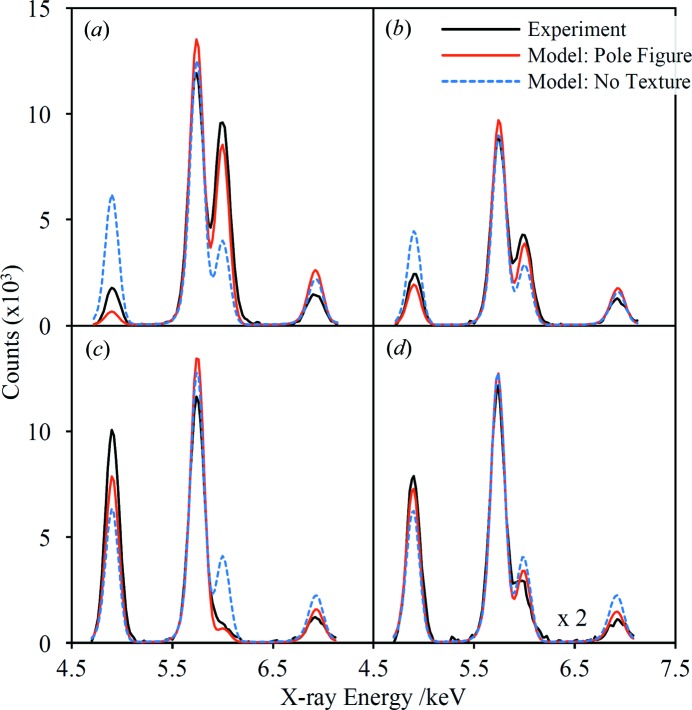
Cu-suppression spectra of CDA510 in the range 4.7–7.1 keV compared with two different model fits. The experimental spectra have been baseline-subtracted and were acquired over one hour. Relative peak intensities in the model fits are determined either by assuming no texture or by extracted pole figure data – see the main text for details. Each fit has just two fitted parameters, an overall scaling factor and the unit-cell size. The diffraction peaks in each spectrum are 220, 311, 222 and 400 in order of increasing energy, and the restricted energy range was chosen because each peak corresponds to one of the four independent pole figures shown in Fig. 11 ▸. The four panels are (*a*) φ = 0°, (*b*) φ = 45°, (*c*) φ = 90°, (*d*) φ = 0° with a 15° sample tilt relative to the surface of the instrument nosepiece (equivalent to χ = 12°). For panels (*a*)–(*c*), the sample surface was flush with the nosepiece, corresponding to χ = 27°. The spectra in panel (*d*) have been multiplied by a factor of two to compensate for the lower count rate.

The major changes in the diffraction peak intensities in Fig. 10 ▸ can be visually correlated with the pole figures in Fig. 11 ▸. For example, the 111 pole figure shows clearly why the 222 diffraction peak is strongest in the φ = 0 and 180° spectra and virtually absent in the φ = 90 and 270° spectra. To enable a quantitative comparison, the texture data encapsulated in the pole figures have been extracted for use in model fits of the HHXRD spectra. Firstly, the data for each pole figure were normalized to units of ‘multiples of a random distribution’ as described by Randle & Engler (2000 ▸). As illustrated in Fig. 11 ▸, the prototype instrument geometry encompasses a range of φ and χ angles in a single measurement because of the moderate collimation of the X-ray beam. By simulating the instrument geometry using ray-tracing software (Hansford, 2009 ▸), it was determined that the measurement includes a 32° interval in φ and a range of χ = 18–35°, with the on-axis ray having χ = 27° when the sample is flush with the nosepiece. The rays are concentrated in the centre of this region and the ray-tracing results were also used to derive an area-weighting function. For each sample orientation, an average value was extracted from the appropriate region of each pole figure and used within the model to modify the calculated peak intensities. Fig. 12 ▸ shows the model results alongside the experimental data, including a data set with a 15° sample tilt which reduces χ to 12°. In each case, the fit that uses pole figure data is a considerable improvement over the texture-free fit.

## 5. Discussion   

The prototype HHXRD instrument described here incorporates some compromises in its design that can be addressed in a purpose-designed instrument. The need to acquire and subtract background spectra can be eliminated by ensuring that the primary X-ray beam lies entirely within the nosepiece aperture. The prototype requires long data-acquisition times compared with the unmodified HHXRF instrument for three primary reasons. Firstly, the X-ray path lengths have been significantly extended and so the relevant X-ray-beam solid angles are much smaller; secondly, diffraction has a lower interaction probability than fluorescence, typically by an order of magnitude or more; and thirdly, the X-ray tube output is much lower because of the reduced excitation voltages. However, there is considerable scope to shorten acquisition times. Reducing the X-ray path lengths as described by Hansford (2011 ▸), with no other changes, would increase count rates by a factor of seven. Replacement of the 10 mm^2^ detector with an annular detector having a total area of 60 mm^2^ (Schlosser *et al.*, 2010 ▸) would bring an additional sixfold reduction in acquisition times. A further near-sevenfold improvement could be achieved through the use of an X-ray tube capable of an emission current of 400 µA in place of the 60 µA limit in the prototype. The much lower excitation voltages required by the back-reflection EDXRD technique relative to XRF applications, up to ∼10 kV, means that 400 µA is achievable given the 4–5 W X-ray tubes commonly specified in modern handheld XRF devices. (The use of lower X-ray energies for EDXRD measurements also means that the radiation shielding employed in HHXRF devices is expected to be effective in an HHXRD instrument.) Exploiting these three changes would bring the 2.5 h acquisition time down to ∼30 s. Full implementation would require careful engineering design and represents a high degree of optimization but is nevertheless feasible. There is also scope to optimize the design of the X-ray tube to maximize *Bremsstrahlung* output by, for example, using a thinner anode layer, switching to a different anode element (see below) or switching to a reflection tube. Furthermore, the signal-to-noise ratios achieved in some of the results presented here, particularly at higher excitation voltages, are greater than strictly necessary. Reduced measurement times will also bring higher detector count rates, but the estimated count rates are expected to be no more than a few tens of thousands of counts per second per detector channel for the majority of samples, well within modern SDD capabilities. Thus, an instrument designer has a certain amount of leeway in achieving short acquisition times to enable true handheld operation.

**Figure 13:**
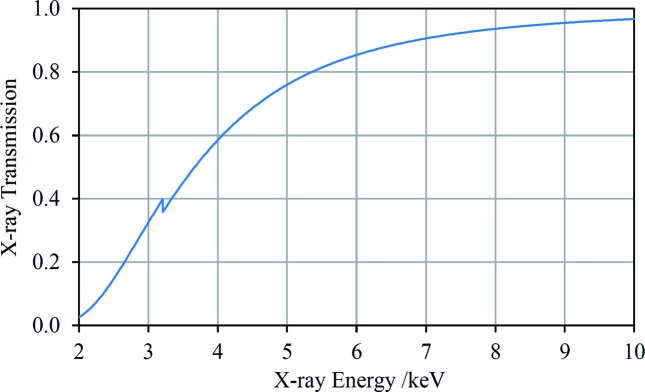
The transmission of X-rays through 55 mm of air and a double pass through a 4 µm polypropyl­ene window (Henke *et al.*, 1993 ▸).

From a performance perspective, it would be best to evacuate the sensor head of an HHXRD instrument to maximize X-ray transmission. Naturally, provision of a diaphragm pump with associated batteries brings a significant penalty for a portable instrument in terms of weight and inconvenience. Assuming a total path length in air of 55 mm [see Table 1 ▸ of Hansford (2011 ▸)] and a 4 µm polypropyl­ene instrument window, the attenuation of X-rays in the range 2–10 keV is shown in Fig. 13 ▸. Avoiding the use of a vacuum pump would allow access to the spectral range down to perhaps 2.5 keV. It would also be advantageous to use an element for the X-ray tube anode with no characteristic lines within the spectral range of interest; possibilities include Mo (with *L*-series lines at ∼2.3 keV) and W (*M*-series lines at ∼1.8 keV, though these lines would interfere with Si XRF quantification). The increase in the spectral range available for crystallographic analysis can only serve to improve the quality of the results. For iron-ore samples, for example, a wider spectral range combined with the reduced density of diffraction peaks towards lower energies (Hansford, 2011 ▸) would allow an improvement in the ability of the instrument to distinguish phases and is also expected to improve the detection limit of any given phase in a mixture. If a vacuum pump is not used then all spectra will contain Ar *K* fluorescence, albeit quite weak. These peaks would have to be included in the analysis, but the benefits of an enlarged spectral range outweigh this disadvantage.

Although the description of a purpose-designed instrument has been focused on XRD performance, such an instrument would retain excellent XRF capabilities. For example, the X-ray tube could be operated at a high excitation voltage in combination with appropriate primary beam filters (Ellis, 2002 ▸) to optimize XRF performance for elemental detection and quantification. Suitable filters would serve to suppress the appearance of interfering diffraction peaks at lower energies, while at higher energies diffraction peaks are heavily overlapped and simply contribute to the background (as in conventional EDXRF). The availability of an XRF analysis would greatly aid the interpretation of XRD results. For example, the identification of XRF peaks due to minor/trace elements within the EDXRD spectral range would aid the processing of diffraction data while elemental quantification would place strong constraints on the phase analysis of the XRD data in some cases. Conversely, identification of the major sample phases would avoid the need to assume the matrix composition in the XRF analysis, leading directly to improved elemental quantification. There is clear potential for the development of a powerful combined XRD/XRF instrument.

The proposed use of an annular detector maximizes the signal while conforming to the angular constraints of the back-reflection EDXRD method and, in this sense, this is the ideal detector. The use of a multi-channel detector would bring additional analytical benefits. For geological samples that have not been prepared by powdering, an indication of crystallite sizes could be derived by assessment of the similarity or otherwise of the spectra acquired by each channel. Major differences would indicate the presence of large crystallites and could be used by the instrument to inform the user that a phase analysis cannot be performed. For smaller differences, summing the spectra would improve powder averaging and reduce sensitivity to preferred orientation. Multiple detectors would also considerably speed up texture analysis by increasing the number of independent measurements for each sample orientation. The disadvantages of multiple detectors are the concomitant increase in instrument mass, which would be moderate, and the higher cost.

The iron-ore results presented in §4.1 show that the HHXRD prototype can distinguish and quantify the composition of samples comprising up to four minerals despite the high degree of peak overlap. In the analysis, the amounts of the four primary minerals were normalized to 100%, necessarily implying overestimation if additional phases are present in the sample. It may not be possible to capture such minerals directly in the phase analysis if they are present in low amounts, especially if they also have relatively featureless diffraction profiles because of low symmetry and/or large unit cells or amorphous content. However, these minerals will leave a signature of one sort or another in the data by contributing diffraction intensity that is not accounted for in the model fit and, ideally, if they also have XRF peaks that cannot otherwise be explained. An XRF analysis would constrain the amounts of minerals not directly detected in the phase analysis, especially when combined with independent knowledge of the minor phases likely to be present in samples, readily available in a mining context. It is expected that considerable progress could be made in disentangling the various sources of uncertainty in the HHXRD analyses, including the goethite and magnetite quantification biases, by focusing on the analysis of prepared mixtures with accurately known compositions.

From an XRD perspective, the iron-ore and limestone (see supporting information) samples are significantly simpler than most geological materials as they are dominated by a small number of minerals with relatively high crystal symmetry. They are also dominated by a small number of elements and so have spectral ranges that are largely free of overlapping XRF peaks. The fluorescence suppression technique (Hansford *et al.*, 2014 ▸) mitigates the interfering effect of XRF peaks but does not eliminate this problem. For these reasons, an HHXRD instrument based on the methods described here will not function as a general-purpose geological field tool. Additional mining applications may be feasible depending on the extent to which the relevant material conforms to the general characteristics described above. If analysis is to be performed on unprepared samples, then it is also necessary that the samples are fine grained. Note that powders are often available at a mine from operations such as drilling of bore holes for explosives.

The results presented in §4.2 show clearly that the limited resolution of the HHXRD prototype is much less of a drawback in the analysis of metallic samples. The phases identified in the EDXRD spectra may all be termed the ‘primary’ phases of each alloy system. Minor phases, which nevertheless are often important in determining material properties, have not been detected with the HHXRD prototype. For example, no carbide phases have been observed for any of the steel samples. As well as having low abundance, secondary phases often have lower crystal symmetry than the primary phase(s) and consequently the diffraction intensity is more distributed across the spectral range, essentially contributing to the background. For alloy systems that commonly consist of only one primary phase, such as Al (f.c.c. α-Al), Ni (f.c.c. γ-austenite) and Cu (f.c.c. α-Cu) alloys, the identification of this phase is unlikely to provide any useful information, although derivation of the unit-cell parameters could be useful for some applications. Conversely, quantification of two-phase systems has much greater potential utility. Examples are Ti alloys (hexagonal close packed α-Ti, body-centred cubic β-Ti), brass alloys (f.c.c. α-Cu/Zn, β′-CuZn) and iron/steel alloys (ferrite/martensite, austenite). Indeed, the measurement and control of the amount of retained austenite is important for many different classes of steel [see, for example, Witte & Lesch (2018 ▸)]. The following discussion focuses on the quantification of retained austenite, but the same principles apply to other two-phase systems. XRD is generally regarded as the most accurate method for retained austenite quantification (Jatczak *et al.*, 1980 ▸) and relies on the comparison of integrated diffraction peak intensities of austenite and ferrite/martensite. For samples with low to moderate texture, the use of the intensities of up to just three peaks in total is suggested in a widely used standard (Jatczak *et al.*, 1980 ▸). For stronger texture, incorporating as many diffraction peaks as possible in the calculation is preferable (Jatczak *et al.*, 1980 ▸; ASTM International, 2013 ▸; Magner *et al.*, 2002 ▸). Accuracy can be improved further by rotating and/or tilting the specimen during data acquisition in order to average-out the effect of texture (Miller, 1968 ▸; Zhang *et al.*, 2000 ▸); this method is also effective for samples with large grain sizes. Regarding HHXRD measurements, data acquisition using Cr suppression yields three resolved austenite peaks and two resolved ferrite peaks (Figs. 8 ▸ and 9 ▸), which is likely to allow sufficient accuracy in many cases. An advantage of EDXRD is that sample rotation/tilting can be slow because the entire spectrum is acquired continuously with no scanning involved. The simplest method to calibrate the retained austenite measurement using EDXRD is through the use of randomly oriented samples with known austenite content (Voskamp, 1974 ▸). An alternative is to calculate theoretical intensity factors (Jatczak *et al.*, 1980 ▸), but the intensity distribution of the source continuum must be measured in this approach. The presence of carbides in the sample can affect the accuracy of the retained austenite calculation *via* potential overlap of diffraction peaks with austenite and ferrite peaks, while the volume fraction of carbides should ideally be included in the calculation (Jatczak *et al.*, 1980 ▸; ASTM International, 2013 ▸). The presence of carbides may limit the achievable accuracy of the retained austenite measurement using the HHXRD methods described here, but further work is needed to understand this limitation. The results presented in §4.2.2 suggest a lower limit of detection of approximately 2 wt% for austenite in stainless steels, improving to below 1 wt% in favourable cases.

The focus within this paper is the implementation of the back-reflection EDXRD technique in a handheld device, but other instrument formats may be appropriate for certain applications such as online analysis in metal production. For example, manufacture of advanced high-strength steels requires close control of phase composition and texture and the back-reflection technique could provide rapid feedback for the fine-tuning of production processes such as heat treatments. A static installation of this type would relax the instrument design constraints, allowing incorporation of a more powerful X-ray source, multiple detectors and an evacuated sensor head as standard. A measurement stand-off distance of a few centimetres is tolerable in these circumstances. Similarly, a benchtop instrument would enable, for example, automated analysis of a series of samples.

The back-reflection EDXRD technique may be particularly suited to quality-control applications involving comparison of samples with a standard specimen. It is easy to envisage an HHXRD device operating in a pass/fail mode, accepting samples that deviate from the standard only within well defined limits for specific diffraction parameters. HHXRD could also be used for presumptive testing, highlighting samples that require more sophisticated analysis with laboratory-based equipment. Similarly, the technique could be used to track the changes in a sample that occur as it ages or during the manufacturing process. The detection of small differences between related samples is illustrated by the results presented in Fig. 9 ▸. The analysis of five 304 stainless steels in §4.2.2 shows the significant crystallographic differences that arise through the application of alternative processing methods and heat treatments to alloys with identical chemistries. A combined handheld XRD/XRF instrument would have the capability not only to determine the grade but also to detect phase composition and texture characteristics that may allow treatment histories to be inferred. These capabilities open up potential applications in quality control and the extension of positive material identification to include treatment history.

The texture measurements by the HHXRD prototype presented in §4.3 are intended as a proof-of-principle demonstration. The next step is to measure complete pole figures and derive the orientation distribution function (ODF) for comparison with a standard analysis. A significant advantage for texture measurements using EDXRD is the simultaneous capture of multiple diffraction peaks (Szpunar & Gerward, 1980 ▸), considerably shortening the total data-acquisition time required to calculate an ODF, especially if multiple detectors are also used. For HHXRD measurements, these comments particularly apply to Cu and Al alloys for which a wide spectral range is typically available (Figs. 5 ▸ and S6). An alternative is to exploit the simultaneous acquisition of several independent texture data points by employing a small number of sample orientations (Szpunar, 1990 ▸) or even with a fixed geometry in order to track dynamic texture changes or for online applications (Gerward *et al.*, 1976 ▸; Bunge *et al.*, 1989 ▸; Kopineck, 1993 ▸). To facilitate texture measurements, a benchtop instrument design would be preferable, incorporating some form of texture goniometer to reorientate the sample relative to the instrument. Ordinarily, prototype HHXRD measurements have been made with samples touching the instrument nosepiece (Fig. 1 ▸); texture measurements require a stand-off distance of perhaps a few centimetres to accommodate sample reorientation. To mitigate the additional X-ray attenuation it would be advantageous to evacuate the sensor head for these measurements. Some limits would naturally be placed on the size and complexity of the sample, as for conventional methods. The prototype instrument has quite limited resolution in φ and χ (see Fig. 11 ▸), and shortening the X-ray path lengths would degrade the angular resolution further. For these reasons, optimization of texture measurement using the back-reflection method requires a dedicated instrument design. A handheld instrument could nevertheless be useful for basic texture measurements such as determining the severity of texture for retained austenite assessment.

Some sample types present particular difficulties even with the application of the fluorescence suppression technique because their primary XRF peaks lie at energies that are otherwise very useful for acquiring diffraction information. Examples are Ca- and Ti-dominated samples such as limestone/dolomitic rocks (§S2) and Ti alloys (§S4). The same problem affects samples with high Cr content, such as Ni alloys and stainless steels, though to a lesser extent. There are two options in these cases. The first is to operate at a sufficiently low accelerating voltage to suppress the offending XRF peaks; the disadvantages are the low *Bremsstrahlung* tube output and the reduced EDXRD spectral range. The second option is to choose a higher setting such as 10 kV and record diffraction peaks above the XRF peaks. In this case, the diffraction peak intensities are typically quite low because of enhanced absorption of X-rays above the elemental absorption edge, and there is also greater overlap of the diffraction peaks because of their higher density (see Fig. S5, for example). For the above examples, the first option was found to be preferable despite the extended acquisition times. A purpose-designed instrument is expected to alleviate these problems considerably because of access to a broader spectral range and tolerable measurement times.

It is clear that the performance of the HHXRD prototype is not competitive with laboratory-based angle-dispersive diffractometers in terms of the accuracy or precision with which crystallographic parameters can be derived or indeed whether an analysis is possible at all. There are, however, three key differentiators enabled by the HHXRD technology presented in this paper. Firstly, measurements can be made *in situ*, bringing the instrument to the sample. In a geological context, decisions can be made at the mine face prior to, for example, blasting operations. In metallurgy, components may be inspected wherever they are located and potentially without having to dismantle assemblies. Secondly, assessments will be available on the timescale of a minute, in complete contrast to common laboratory response times of days or weeks. Thirdly, the method accommodates curved and irregular surface morphologies, and analyses can be conducted with no sample preparation for many mining and a large majority of common alloy samples. The performance of HHXRD based on back-reflection EDXRD is not sufficient to provide a general-purpose XRD tool either for geological or for metallic samples. However, the prototype instrument results reported here provide support for the effectiveness of HHXRD in targeted applications. The chances of successful analysis are greater for simpler applications with well defined, specific aims such as determination of retained austenite in steels. Independent ancillary information can also enable meaningful analyses, such as provision of a short list of the minerals that may be present in mining samples, specific to each mine. In metals production, most of the material characteristics will be known in advance for well established processes, and a simple measurement may be sufficient for quality control or for fine-tuning of the process. For such applications, HHXRD could become the method of choice given its speed and convenience.

Other application areas are feasible and a particularly interesting example is the field of cultural heritage (Hansford *et al.*, 2017 ▸). Naturally, a very wide range of materials are encountered in the field as a whole and the use of HHXRD would need to be targeted to specific material types such as metallic artefacts. The application of nondestructive techniques is increasingly important in archaeometry studies (Young, 2012 ▸; Janssens *et al.*, 2016 ▸) and instrument portability is also a crucial advantage (Chiari, 2008 ▸). Curators are reluctant to allow artefacts to leave the museum/collection and HHXRD devices could also be taken to archaeological sites to improve conservation decisions. A relatively low ‘hit rate’ of successful *in situ* XRD analyses may be more acceptable in heritage studies than in other fields.

## 6. Conclusions   

There are several key factors unique to back-reflection EDXRD that enable the development of a true handheld XRD instrument. Short measurement times can be achieved because the geometry favours the capture of the diffracted intensity of whole Debye–Scherrer rings (which also reduces sensitivity to texture) and because the technique allows the capture of a relatively wide range of 2θ angles with only a minor penalty in terms of peak broadening (Hansford, 2011 ▸). Furthermore, the insensitivity of the technique to sample morphology and the precise instrument-to-sample distance implies that a handheld instrument will be tolerant to minor operator movements relative to the sample during data acquisition. The lack of a sample preparation requirement in many cases also saves considerable time and is highly convenient, and the back-reflection geometry favours a compact and lightweight design. The maturation of technologies for HHXRF (Bosco, 2013 ▸) is enabling for the development of HHXRD based on EDXRD methodology.

The prototype instrument results show significant promise for the application of HHXRD in the fields of mining and metallurgy. Such a device does not have technical performance characteristics that can even approach those of laboratory equipment. However, it is expected that the capabilities of a purpose-designed instrument would be sufficient to prove commercially useful for targeted applications such as the mineralogical analysis of iron-ore samples and the quantification of retained austenite in a broad range of steels. The benefits of rapid, *in situ* and nondestructive analysis, coupled with no requirement to prepare samples in the majority of cases, have the potential to be transformative for applications such as these. Successful implementation of HHXRD requires the development of algorithms capable of producing actionable answers in the field, important for handheld analytical devices in general (Gardner & Green, 2013 ▸; Crocombe, 2018 ▸). Although the design of an HHXRD instrument must focus on optimizing XRD performance, it would be essentially straightforward to incorporate excellent XRF capabilities comparable to those of existing HHXRF devices. The complementarity of XRF and XRD improves the utility of a combined handheld XRD/XRF instrument.

## Supplementary Material

Contains additional results which may be of interest to some readers.. DOI: 10.1107/S1600576718012943/jo5043sup1.pdf

## Figures and Tables

**Table 1:** Iron-ore quantification

Sample†	Hematite‡	Goethite‡	Magnetite‡	Quartz‡	Other minerals	Amorphous fraction§	Rayleigh scale factor	Exposure time¶ (10^3^ s)
A	14.0 (4.6)	30.8 (7.0)	0	55.2 (12.7)	0	–	0.55	8.62
	16.1 (2.6)	27.9 (3.2)	3.4 (1.5)	52.7 (4.0)				
B	36.6 (11.8)	17.4 (6.7)	19.2 (5.6)	26.8 (8.6)	0	–	0.33	9.14
	35.9 (3.5)	12.8 (2.4)	25.2 (3.1)	26.1 (3.1)				
C	49.2 (11.4)	24.9 (5.8)	16.6 (3.2)	9.2 (7.6)	0	–	0.23	9.44
	46.2 (3.8)	21.1 (2.9)	22.1 (3.0)	10.7 (2.3)				
D	10.7 (6.6)	12.5 (7.7)	41.8 (12.4)	35.0 (10.3)	13.9 (2.5)	–	0.60	7.11
	11.2 (2.3)	0	47.4 (3.9)	27.5 (3.2)				
E	42.8 (5.1)	17.3 (5.0)	40.0 (4.8)	0	1.1 (1.0)	–	0.09	10.13
	44.1 (3.8)	5.7 (1.8)	47.6 (3.9)	1.4 (1.1)				
ASCRM 030	26.7 (4.9)	67.8 (12.5)	5.5 (2.5)	0	2	16	0.35	8.55
	26.0 (3.1)	56.0 (4.1)	0	1.0 (1.0)				
ASCRM 031	43.7 (6.0)	52.1 (7.2)	4.2 (2.3)	0	2	3	0.24	9.23
	48.0 (3.9)	47.0 (3.8)	nr	nr				
ASCRM 032	70.3 (15.5)	25.0 (5.4)	4.7 (2.4)	0	5	1	0.23	9.28
	78.0 (4.6)	16.0 (2.6)	nr	1.0 (1.0)				
ASCRM 033	20.8 (5.2)	72.6 (18.4)	6.6 (2.7)	0	2	10	0.31	8.83
	23.0 (3.0)	65.0 (4.3)	nr	1.0 (1.0)				
ASCRM 034	55.8 (8.8)	39.5 (6.2)	4.7 (2.6)	0	4	9	0.26	8.96
	56.0 (4.1)	31.0 (3.3)	1.0 (1.0)	1.0 (1.0)				
ASCRM 035	57.5 (11.1)	36.2 (6.9)	6.2 (3.1)	0	7	6	0.25	9.08
	59.0 (4.2)	25.0 (3.1)	3.0 (1.5)	2.0 (1.3)				

† The ArcelorMittal samples are designated A–E.

‡ Amounts are given underlined for the HHXRD analysis and in normal text for the laboratory analysis with error estimates in parentheses. HHXRD errors are as reported by the least-squares fitting routine and laboratory errors are calculated as (wt%)^0.35^, as specified by Hillier (2016 ▸), for samples A–E and assumed to be the same for the ASCRM samples. An ‘nr’ (not reported) entry indicates identification of the phase but quantification below 0.5 wt%.

§ The amorphous fraction was not determined for the ArcelorMittal samples.

¶ The fitted exposure time is reported. Actual exposure times were 9000 s in each case.

**Table 2:** Copper alloys

Alloy designation†	Description	Elemental composition‡ (wt%)
CDA110	Electrolytic tough pitch	Min. 99.9 Cu
CDA360	Free-cutting brass	Cu 60–63, Pb 2.5–3.0, Balance Zn
CDA510	Phosphor bronze	Sn 3.5–4.9, P 0.03–0.35
CDA614	Aluminium bronze	Al 6.0–8.0, Fe 1.5–3.5
CDA655	High-silicon bronze	Si 2.8–3.8, Mn 0.5–1.3
CDA715	Cupronickel 70/30	Ni 29–33, Fe 0.4–1.0

† Copper Development Association Inc. The equivalent UNS (unified numbering system) designation for CDAxxx is Cxxx00; the last two digits can also be used to specify further sub-divisions.

‡ Balance Cu unless otherwise specified. Additional elements may have specified maximums.

## Footnotes

1 All absorption-edge energies and fluorescence transition energies have been taken from the work by Deslattes *et al.* (2003 ▸).
