# Supplementary material for: A prototype handheld X-ray diffraction instrument
Source: J Appl Crystallogr. 2018 Oct 25;51(Pt 6):1571–85. doi: 10.1107/S1600576718012943 (PMC6276277; doi:10.1107/S1600576718012943)
Supplement: Supplementary file 1 [file j-51-01571-sup1.pdf]

JOURNAL OF
APPLIED
CRYSTALLOGRAPHY

Volume 51 (2018)

Supporting information for article:

A prototype handheld X-ray diffraction instrument

Graeme Hansford

A prototype handheld X-ray diffraction instrument: Supporting Information

1. Iron Ore: Additional Plots

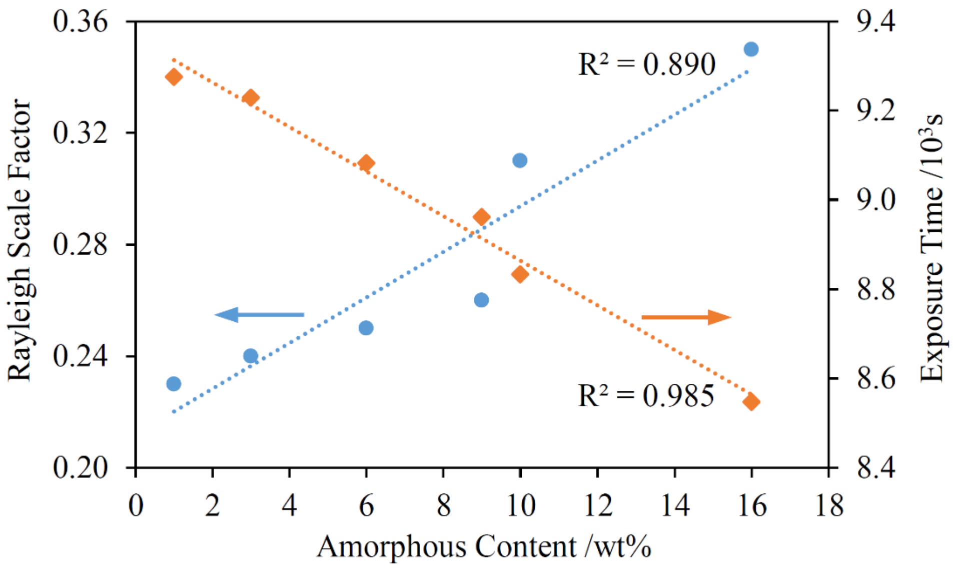

Fig. S1. The Rayleigh scattering scale factor (blue) and the exposure time (orange) plotted against the amorphous content of the ASCRM samples (all data taken from Table 1). R^2 values show good correlations in both cases, especially exposure time versus amorphous fraction.

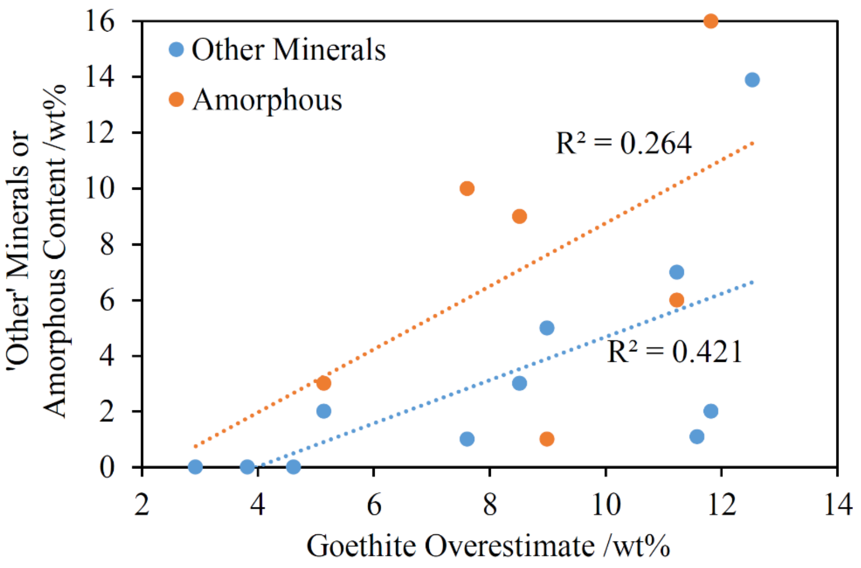

Fig. S2. The amount of 'other' minerals and the amorphous fraction plotted against the overestimate of goethite by the HHXRD prototype relative to laboratory quantification (all data taken from Table 1). There is a degree of correlation between 'other' minerals and the goethite overestimation but quite weak correlation with amorphous content.

2. Limestone and Dolomite Rock

The prototype instrument was tested with seven limestone and dolomite rock samples in order to assess the capability of the device to identify and distinguish calcite ($CaCO_3$) and dolomite [$CaMg(CO_3)_2$]. Six of the samples were unprepared rock specimens and one was a pressed powder pellet of the Japanese geological standard JDo-1 (Imai, N., Terashima, S., Itoh, S. & Ando, A. [1996]. *Geostand. Newsl.* **20**, 165–216). Three of the rock specimens are the same as reported earlier (Hansford *et al.*, 2014); full details are given in the figure caption. The X-ray tube was operated at an excitation voltage of 4 kV in order to suppress Ca-K fluorescence. Working at such a low voltage has the disadvantage that the tube emission intensity is very low and the spectrum for each sample was acquired over five hours to compensate. Another disadvantage is that the spectral range over which diffraction peaks can be observed is significantly smaller than for the iron ore samples, and so for this set of samples the Rh-L region has been included in the assessment and no attempt has been made at quantification, though most of the samples appear to be dominated by one mineral in any case. For these datasets the background spectrum was subtracted but the energy scales have not been re-calibrated because of a lack of suitable XRF peaks. A small correction has been made for the presence of Ar-K fluorescence in the background spectrum, as described in §3. The background spectrum has a higher intensity at the Rh-Lα energy (2.7 keV) than most of the sample spectra resulting in negative-going data in the final spectra. The same effect has been observed for many metallic samples, §4.2, and possible explanations are discussed in §S7.

The spectra in Fig. S3 are separated into calcite- and dolomite-dominated samples. The model reproduces the experimental data quite well, with the largest discrepancies for diffraction peaks enhanced by Rh-L lines. Comparing the experimental datasets, there is a high degree of consistency within each subset, setting aside the XRF peak intensities for which variability is expected. The diffraction peaks enhanced by overlap with Rh-L lines show a greater variability. This effect may possibly reflect small shifts in the diffraction peak positions due to variation in the unit cell parameters of calcite and dolomite because of the propensity of these minerals to accommodate solid solutions (see for example, Zhang, F., Xu, H., Konishi, H. & Roden, E. E. [2010]. *Am. Mineral.* **95**, 1650-1656). It also highlights why this spectral region is normally neglected in the analysis of prototype HHXRD data. Clearly, the instrument can distinguish calcite and dolomite in samples that are dominated by either one of these minerals. Sample A is known from previous work (Hansford *et al.*, 2014) to contain approximately 14% dolomite, and indeed there is a peak in the corresponding spectrum of this sample consistent with the presence of dolomite (indicated in Fig. S3), as well as a small Mg XRF peak.

The analysis of limestones and dolomite rocks presents a simpler scenario than the analysis of iron ores because these rock types are dominated by just one or two minerals (calcite and dolomite). A purpose-designed instrument would be expected to perform significantly better than the prototype by eliminating the interfering Rh-L lines. There is every reason to suppose that quantification of samples dominated by calcite and dolomite would be straightforward (see also Hansford *et al.*, 2014).

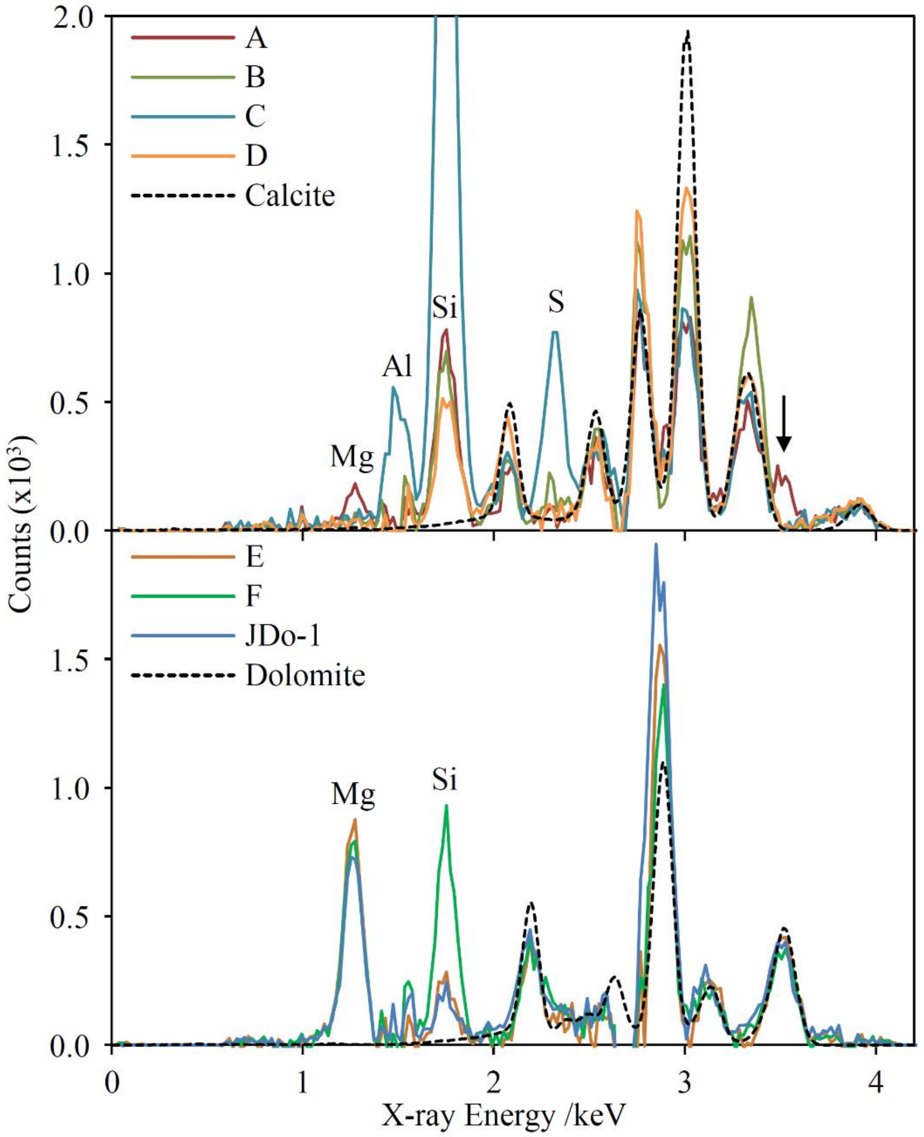

Fig. S3. Prototype HHXRD spectra for samples dominated by calcite (top panel) or dolomite (bottom panel) along with model simulations of each mineral. XRF peaks are labelled with the corresponding element; the calcite diffraction peak at 3.3 keV for sample B may be overlapped by a K-Kα fluorescence peak. The Rh-Lβ_2 peak at 3.00 keV is enhanced by overlap with a calcite diffraction peak while the Rh-Lβ_4 peak at 2.89 keV is enhanced by a dolomite peak. Some other Rh-L lines overlap diffraction peaks but with much lower enhancements; simulations include the Rh-L line enhancements by diffraction peaks. The arrow in the top panel shows a dolomite diffraction peak for sample A. The samples were sourced from the following geological locations: A: St Louis, Missouri, USA; B: Hope Valley, Peak District, UK; C: Agios Petros beach, Alonissos, Greece; D: chalk of unknown origin; E: Mindelheimer Klettersteig, Allgäu Alps, Austro-German border; F: dolomite rock of unknown origin.

3. Nickel Alloys

Seven Ni alloys were analysed with the HHXRD instrument; see Table S1 for details of the alloys including elemental composition. Ni alloys typically incorporate significant quantities of other transition series elements, notably Cr, Fe, Co and Cu. The first three of these elements have K-series fluorescence peaks present in the spectrum if the X-ray tube is operated at 8.3 kV to suppress Ni-K

fluorescence. Although diffraction peaks are observed in these spectra, often as shoulders on the XRF peaks and sometimes as resolved peaks, a more reliable method to obtain EDXRD spectra relatively free of XRF peaks is to use Cr-suppression (tube voltage 5.9 kV), see Fig. S4. All of the spectra show the presence of the primary γ-austenite fcc phase with no secondary phases identifiable for any sample. Each diffraction peak in Fig. S4 shows variation in position as a consequence of differences in unit cell dimensions which can be extracted from the HHXRD data as for the Cu alloys (see §4.2.1). Model simulations suggest that intensity variations mainly reflect differences in texture with only a minor contribution from compositional differences.

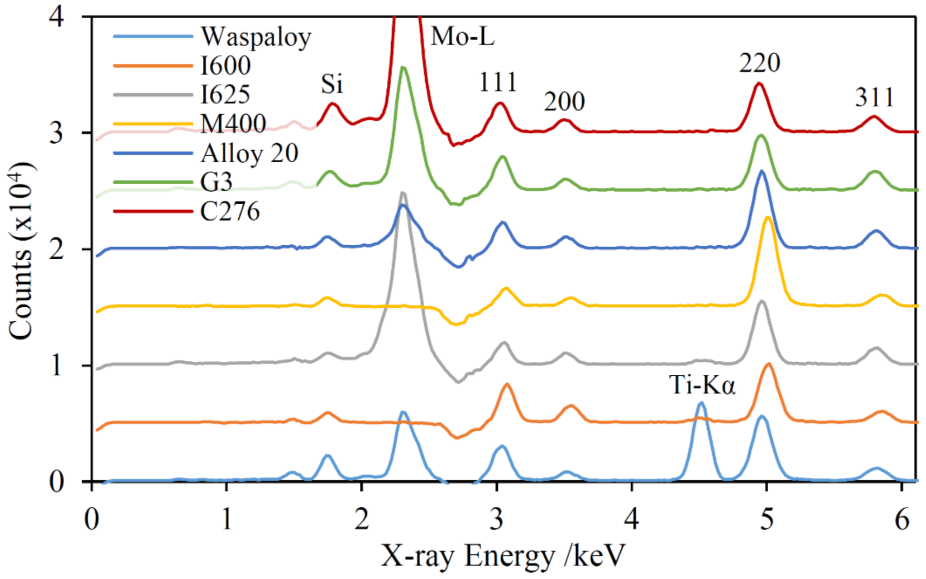

Fig. S4. The Cr-suppression spectra of the seven Ni alloy samples. XRF and diffraction peaks are labelled with the corresponding elements and Miller indices respectively.

Table S1 Nickel alloys.

Common Name	UNS Designation	Elemental Composition[a]										
		Ni	Al	Ti	Cr	Mn	Fe	Co	Cu	Nb	Mo	W
Waspaloy	N07001	59.0	1.1	3.1	19.7	-	0.8	12.2	-	-	4.0	-
Inconel 600	N06600	74.4	-	-	15.9	0.2	8.9	0.1	0.2	-	-	-
Inconel 625	N06625	65.2	-	0.2	17.5	0.3	4.0	0.3	0.1	3.4	8.5	0.2
Monel 400	N04400	64.5	-	0.1	0.3	1.5	1.8	-	31.3	-	0.3	-
Alloy 20	N08020	33.6	-	-	18.7	0.6	40.9	-	3.2	0.4	2.1	-
Hastelloy G3	N06985	47.9	0.3	-	20.1	0.7	18.3	1.9	1.7	0.2	7.3	1.0
Hastelloy C276	N10276	56.7	0.8	-	14.6	0.5	6.5	1.3	0.2	-	15.7	3.2

[a]Elements with at least 1.0 wt% for at least one alloy are specified. A null entry indicates < 0.1 wt%.

4. Titanium Alloys

Three Ti alloys were analysed, one grade 2 (UNS R50400) commercially-pure alloy and two grade 5 alloys (UNS R56400, also known as Ti6Al4V). For each sample, data was acquired operating the HHXRD instrument at 4.9 kV to suppress Ti-K fluorescence and at 10 kV to access a wider spectral range; the resulting spectra are shown in Fig. S5. It is apparent from these spectra that Ti alloys present a greater challenge to HHXRD analysis than many other metallic samples. The Ti-K fluorescence peaks lie at 4.5 – 5.0 keV which is squarely within the range typically yielding useful diffraction data for other samples. Operating the instrument at a sufficiently low excitation voltage to suppress these fluorescence peaks gives rise to a small useful spectral range, ~3.3 to 4.9 keV, and relatively poor signal-to-noise ratios because of lower tube emission intensity. Only three diffraction peaks of the α-Ti phase (hexagonal close-packed, hcp) occur within this range. At the higher excitation potential, the very strong Ti-K peaks (and, for the common Ti6Al4V alloy, the V-K fluorescence peaks also) together with the low-side tails and escape peaks result in no useful diffraction information below ~5.7 keV. Furthermore, the diffraction signals above the Ti-K absorption edge at 4.965 keV are relatively weak because diffraction is competing with strong absorption by Ti. There is also a contribution to the intensity in the range 9 – 10 keV from pile-up of the fluorescence peaks. Lastly, the α-Ti phase belongs to the hexagonal crystal system leading to a higher number of diffraction lines and greater peak overlap than for metals with cubic symmetry. The peak density hampers the usual data processing step of fitting and subtracting the scattered-intensity baseline for the 10 kV spectra.

Despite these difficulties some simple conclusions can be reached regarding the Ti alloy samples. All three are dominated by the α-phase. One of the Ti6Al4V samples has a weak peak at 3.9 keV that indicates the probable presence of the bcc β-phase at the ~10% level. Model fits of the 10 kV dataset for this sample are consistent with this conclusion but with a lower level of confidence – the Ti-suppression dataset gives the best evidence for the β-phase. Independent laboratory XRD analyses have identified the presence of the β-phase in both the Ti6Al4V samples. Rietveld analyses were performed for these samples and suggest that the Ti6Al4Va and b samples contain 1% and 6% β-phase respectively, though the presence of texture hampered the analyses. Nevertheless, the HHXRD results for these two samples are certainly consistent with the independent analyses. The grade 2 sample was not independently analysed. The marked differences in relative diffraction peak intensities between the samples strongly suggests major differences in their texture characteristics. Pawley fits to the datasets can be used to extract unit cell parameters, but the accuracy is reduced relative to other metals because two unit cell parameters are fitted instead of one and because of peak overlap.

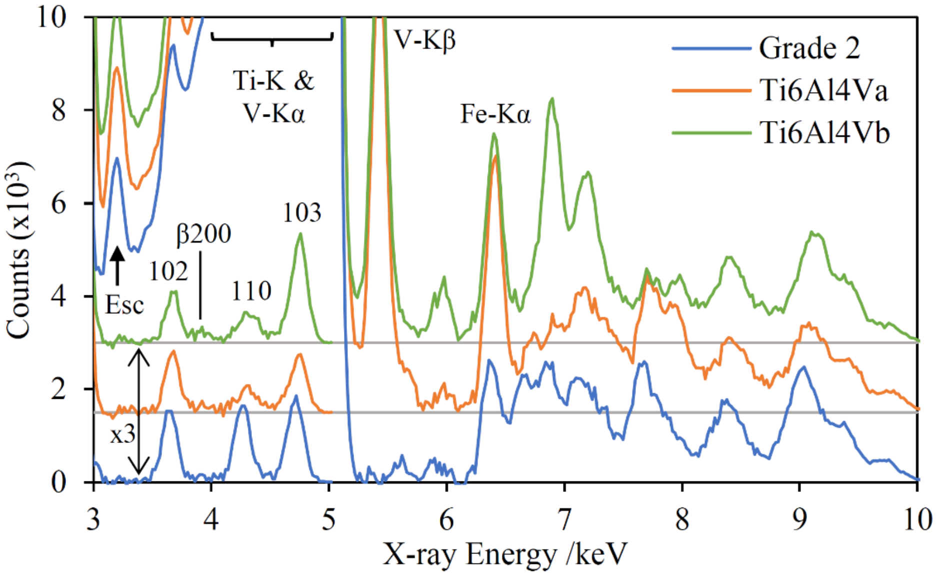

Fig. S5. The Ti alloy prototype HHXRD spectra acquired using Ti-suppression (5 hr datasets, multiplied by a factor of 3 prior to plotting) and 10 kV excitation voltage. Ti6Al4Va and b were supplied by Goodfellow Cambridge Ltd (Huntingdon, UK) and PMI Analytical respectively. No baseline subtraction was performed for the 10 kV datasets which have been plotted on a vertical scale appropriate for the diffraction peaks >6 keV. The spectra have been offset vertically and the horizontal lines show the zero levels for the offset spectra. XRF peaks have been labelled and 'Esc' denotes the Ti-Kα escape peak. The Ti-suppression diffraction peaks have been labelled with the α-Ti phase Miller indices and the weak peak assigned to 200 of the β-Ti phase is also indicated.

5. Aluminium Alloys

A set of seven standard-grade Al alloys were analysed, two of which were nominally-identical 6061 (T6 temper) grade but from different suppliers. Six of the samples were part of the alloy check sample kit and one of the 6061 samples was supplied by Goodfellow; details of each alloy are given in Table S2. Al is the lightest base element of the alloy systems tested in this study and the Al-K XRF peaks, at ~1.5 keV, lie well below the spectral range that is useful for the observation of diffraction peaks. A tube excitation voltage of 10 kV was used to acquire most of the spectra, Fig. S6. Al is typically alloyed with no more than 10 wt% of additional elements, and transition series elements with K-series XRF peaks potentially overlapping the diffraction peaks, such as Cr, Mn, Fe and Cu, are often present at <1 wt%. In these cases the XRF peaks have comparable intensity to, or are weaker than, the diffraction peaks and can be accommodated in model fits to the data. Two of the grades, 2024 and 7075, have >1 wt% alloyed Cu and for these samples Cu-suppression data was preferred and the corresponding spectra are shown in Fig. S6. When subtracting the background spectrum for each of the Al alloy datasets it was noted that the Ti-Kα XRF peak arising from the vacuum window overlapped the much weaker 220 diffraction peak at ~4.4 keV. This peak was therefore excluded from the fits: the spectral range 4.75 – 10 keV (4.75 – 9 keV for Cu-suppression) was used in the analyses.

All of the samples clearly show the α-Al fcc matrix phase and no secondary phases could be identified in any of the spectra. The unit cell dimensions were extracted for the α-phase, though the variation across the seven samples was quite small, ranging only from 4.053 Å (grade 1100) to 4.060 Å (grade 7075). Variation in the relative intensities of the diffraction peaks between samples are indicative of texture differences, though the effect of XRF intensity variation must be accounted for

when comparing the spectra in Fig. S6. The two 6061 grade samples show only minor differences in their corresponding spectra.

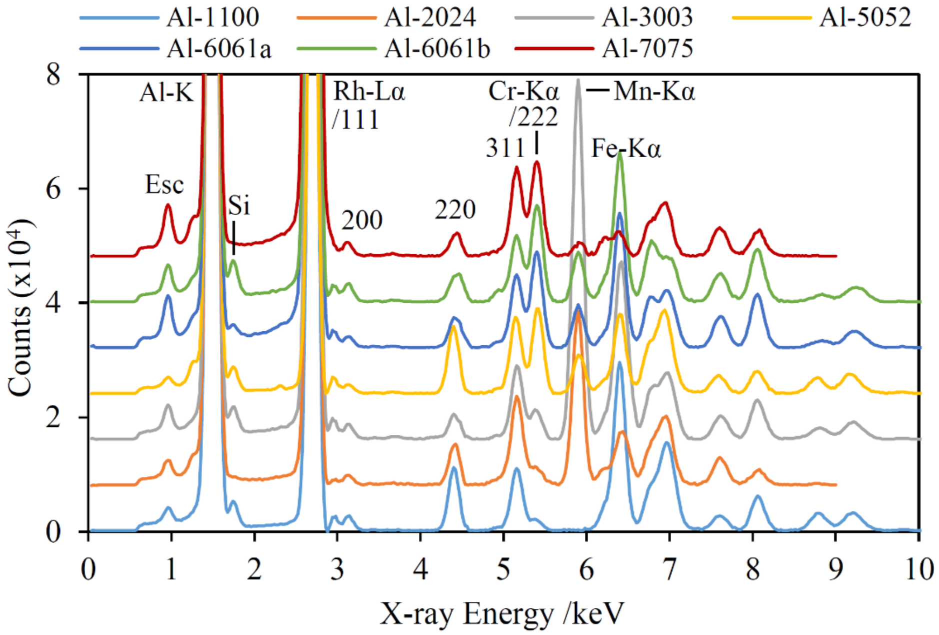

Fig. S6. The prototype HHXRD spectra of seven Al alloy samples acquired using either 10 kV X-ray tube excitation or using Cu-suppression (Al-2024 and Al-7075). The latter datasets have been multiplied by a factor of 1.5 to compensate for the lower tube Bremsstrahlung output. XRF peaks and some of the diffraction peaks have been labelled. Note that the 111 peak is enhanced by overlap with the Rh-Lα tube emission line whereas the 222 peak simply overlaps the Cr-Kα XRF peak. 'Esc' denotes the Rh-Lα/111 escape peak. The weak peak just below 3.0 keV visible in most of the spectra is due to pile-up of the Al-K fluorescence peak. Al-6061a is the Goodfellow-supplied sample.

Table S2 Aluminium alloys.

Alloy Designation[a] (Temper)	Common Uses	Elemental Composition[b] (wt%)
Al-1100 (O)	Universal	Min. 99.0 Al<br>**Cu** 0.05 – 0.20<br>Si+Fe 0.95 max
Al-2024 (T3)	Aerospace	**Cu** 3.8 – 4.9<br>**Mg** 1.2 – 1.8<br>Mn 0.30 – 0.9<br>Si,Fe 0.50
Al-3003 (H14)	Sheet, Structural	**Mn** 1.0 – 1.5<br>Cu 0.05 – 0.20<br>Si 0.6, Fe 0.7
Al-5052 (H32)	Marine, Aerospace	**Mg** 2.2 – 2.8<br>Cr 0.15 – 0.35<br>Si 0.25, Fe 0.40
Al-6061 (T6)	Universal, Structural	**Mg** 0.8 – 1.2<br>**Si** 0.40 – 0.8<br>Cu 0.15 – 0.40<br>Cr 0.04 – 0.35<br>Fe 0.7
Al-7075 (T6)	Aerospace	**Zn** 5.1 – 6.1<br>Mg 2.1 – 2.9<br>Cu 1.2 – 2.0<br>Cr 0.18 – 0.28<br>Si 0.40, Fe 0.50<br>Mn 0.30

[a]The Aluminum Association. The equivalent UNS designation for Al-xxxx is A9xxxx.
[b]Balance Al unless otherwise specified. The principal alloying element(s) for each grade are shown in bold. Additional elements may have specified maximums.

6. Distinguishing Ferrite and Martensite in Steel Samples

Ferrite has a bcc structure and martensite has the closely-related body-centred tetragonal structure, incorporating interstitial C atoms in a supersaturated solid solution. Martensite has diffraction peaks that split relative to the corresponding ferrite peaks, with the details depending on the Miller indices: *hhh* peaks do not split, *hhl* peaks split into two components and *hkl* peaks split into three ($h \neq k \neq l$). The magnitude of each splitting depends on the differences between the indices and between the *a* and *c* unit cell dimensions, but in any case remain relatively modest because of the small difference between *a* and *c* e.g. $a \cong 2.86$ Å, $c \cong 2.96$ Å would be typical for steels with 0.8 wt% C. The split peaks cannot be resolved using the HHXRD prototype and the primary effect is a minor broadening of the diffraction peaks. An example is shown in Fig. S7 which presents the Cr-suppression spectra for EN31 bearing steel[1] (UNS G52986) subjected to two different heat treatments (see the figure caption for details). Overplotting of the spectra shows a broadening of the martensite peaks on the low-energy side, relative to the ferrite 200 and 220 peaks, consistent with model results (the 110 peak is complicated by overlap with the austenite 111 peak). The occurrence of ferrite in the –AC sample and

[1] The author would like to thank Dr Rob Thornton (University of Leicester, UK) for the provision of these EN31 samples.

martensite in the –QT sample has been independently verified using a Bruker D8 Advance diffractometer.

Demonstration of martensite peak broadening, using the HHXRD prototype, is relatively straightforward for two samples that are otherwise very similar and with independent confirmation. For other ferritic/martensitic samples, trial fits of each phase separately can sometimes give a strong indication that one of these phases is dominant in the sample through a significantly smaller χ^2 goodness-of-fit parameter and, in the case of martensite, realistic values for the difference between the *a* and *c* unit cell parameters. However, further work is required to determine whether HHXRD can reliably distinguish these two phases.

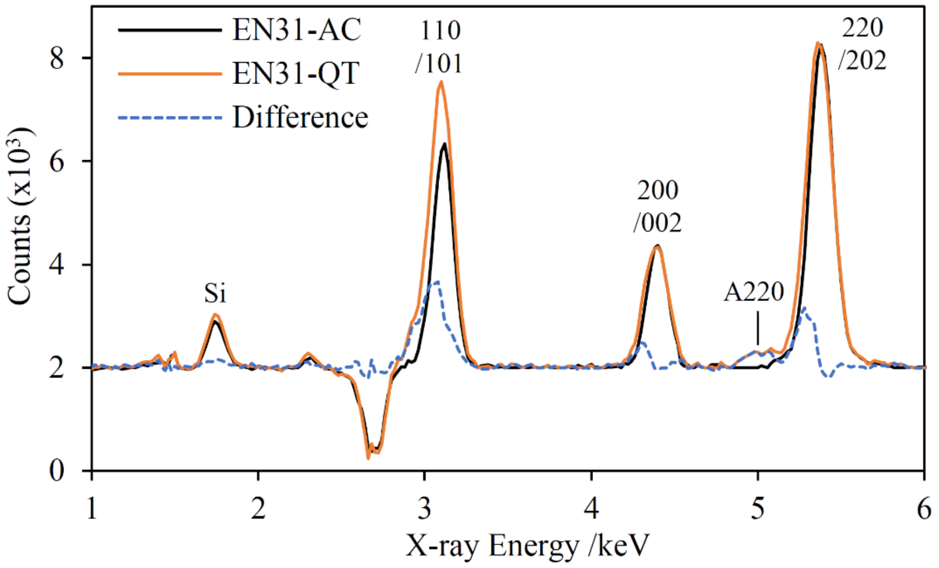

Fig. S7. Cr-suppression spectra of EN31 steel subjected to different heat treatments: AC – air-cooled and cryogenically treated; QT – quenched and tempered. The diffraction peaks are labelled with pairs of indices corresponding to martensite; the first set of indices in each pair is correct for ferrite. The EN31-QT data has been multiplied by 1.2 so that the 200/002 peaks of the two samples have the same height; the scaling works well for the 220/202 peaks as well. The EN31-QT sample has a minor amount of austenite and the 220 peak labelled.

7. Discussion of Negative-Going Peaks in Background-Subtracted Spectra

The scattered Rh-L lines are more intense in the background spectra than in many of the sample spectra, typically by a few percent. Subtraction of the background therefore gives rise to a negative-going peak at ~2.7 keV corresponding to Rh-Lα. It is presumed that the effect is most pronounced at this energy because the Lα line is the most intense X-ray tube characteristic line. Taken at face value, it appears that the Rh-L lines scatter more strongly off air than off solid samples. A similar effect is not seen for the Al-K fluorescence peak due to exposure of the inside surface of the nosepiece, or for the Ti-K fluorescence peaks which are due to trace amounts of Ti in the vacuum window (the intensity of the Ti-K peaks changes significantly when the window is changed). These fluorescence peaks are accurately eliminated by background subtraction. As a result of low count rates, detector deadtime corrections are typically well below 1% and possible inaccuracies in these corrections do not provide a convincing explanation of the negative-going peaks. The enhanced L-line scatter in the background spectra appears to be a genuine physical effect, not a result of a detector or instrument artefact. The effect is masked if the sample has diffraction or fluorescence peaks at the same energy

but otherwise it is observed for all sample types. The scattered Rh-L lines are broadened and shifted to lower energies when compared with a simulation of Rh-L fluorescence peaks. A fit to the scattered peaks yields a value of -19 eV for the shift (for the Ni-suppression background) while theory predicts a Compton shift of -27 eV at 2.7 keV for the geometry of the HHXRD prototype. These values, along with the broadening, strongly suggest that both Compton and Rayleigh scattering contribute significantly to the observed peaks. Comparison of background and sample spectra in the Rh-L region shows very small differences in the overall profile, other than intensity, and the negative-going data is not explained by a differential shift of the Rh-L peaks.

A possible physical explanation of the above effect has been identified. The scattering of X-ray photons by a sample competes with other interaction processes such as absorption. If absorption is very strong then comparatively little scattering will take place. However, the density of air is so much lower than a solid sample that most X-ray photons at 2.7 keV will travel a few cm before interacting (the 1/*e* distance is 3.8 cm) and the majority of scattering events will take place outside the field of view of the detector. Some trial simulations of Rayleigh scattering back up these conclusions. Calculations including Compton scattering are required to make progress in resolving this issue, but the physical origin of this effect remains unclear at present.
